# Systematics of the Phyllognathopodidae (Copepoda, Harpacticoida): re-examination of *Phyllognathopus* viguieri (Maupas, 1892) and *Parbatocamptus jochenmartensi* Dumont and Maas, 1988, proposal of a new genus for *hyllognathopus bassoti* Rouch, 1972, and description of a new species of *Phyllognathopus*

**DOI:** 10.3897/zookeys.104.763

**Published:** 2011-06-13

**Authors:** Diana M. P. Galassi, Paola De Laurentiis, Barbara Fiasca

**Affiliations:** Dipartimento di Scienze Ambientali, University of L’Aquila, Via Vetoio, Coppito, 67100 L’Aquila, Italy

**Keywords:** Harpacticoida, Phyllognathopodidae, taxonomy, *Phyllognathopus*, *Parbatocamptus*, *Neophyllognathopus bassoti* comb. n.

## Abstract

The family Phyllognathopodidae (Crustacea, Copepoda, Harpacticoida) is heavily affected by the floating taxonomic status of the type-genus *Phyllognathopus*. A revision of the different character states displayed by members of the family is presented, and new phylogenetically informative characters are described, enlarging the analysis to the remaining genera of the family, *Parbatocamptus* and *Allophyllognathopus*. *Phyllognathopus viguieri* (Maupas, 1892) and *Parbatocamptus jochenmartensi* Dumont and Maas, 1988 are redescribed in detail, and *Phyllognathopus inexspectatus* **sp. n.** is described from ground water in Italy. The new genus *Neophyllognathopus* is established to accommodate *Phyllognathopus bassoti* Rouch, 1972,originally collected from Long Island (Papua - New Guinea), and subsequently recorded also from the Bantayan Island (Philippines), and from the Indian subcontinent. The new genus is presently monotypic and is easily defined by the unique construction and morphology of leg 5 in both male and female, of male leg 6, and by the peculiar ornamentation of male third and fourth urosomites. Biogeographical and ecological considerations are presented for members of the family.

## Introduction

The harpacticoid family Phyllognathopodidae exhibits a low diversity, currently containing 13 species according to Boxshall and Halsey (2004), accommodated in three genera: *Phyllognathopus* Mrázek, 1893, *Allophyllognathopus* Kiefer, 1967 and *Parbatocamptus* Dumont and Maas, 1988. Members of the family are predominantly recorded from semi-terrestrial and freshwater habitats (i.e. phytotelmata, leaf litter, epibenthic habitats in streams and springs, hyporheic habitats, ground water) (Reid 2001), and findings in anchialine caves or brackish ground water (Huys et al. 1996, pers. comm.; Bruno and Cottarelli 1999) represent mere exceptions.

The family is undoubtedly monophyletic, being instantly recognizable by the unique phyllopodial lamelliform maxilliped, in conjunction with the first pedigerous somite not being incorporated into the cephalosome. Despite the low diversification in the family, the taxonomic history of the type-genus *Phyllognathopus* has been and still is highly controversial (Glatzel and Königshoff 2005). The root cause for this state of affairs lies in three questionable assumptions surrounding the taxonomy of *Phyllognathopus viguieri* (Maupas, 1892) and which were employed early in delimiting species within the genus: 1) its cosmopolitanism; 2) its ecological plasticity, enabling itself to colonize virtually any kind of habitat, from the truly aquatic to semi-terrestrial; and 3) as a reflection of the latter, its morphological variability. The taxonomic confusion surrounding the type-species of *Phyllognathopus* is also reflected in the entire systematics of the family (Boxshall and Halsey 2004, Wells 2007), preventing any reconstruction of the phylogenetic relationships among members of this family.

Recent discoveries of new phyllognathopodid representatives in Italian ground water, together with the re-examination of different species and populations coming from several localities world-wide, allowed a re-analysis of key-taxa within the family, the description of a new stygobiotic species of *Phyllognathopus* and the establishment of a new taxonomic rank for *Phyllognathopus bassoti* Rouch, 1972, assigned herein to the new genus *Neophyllognathopus*.

## Material and methods

Specimenswere collected from hyporheic habitats by using the Bou-Rouch method (Bou and Rouch 1967) and filtering through a 60 µm mesh net. Epibenthic samples from springs were taken by washing sediments with a hand net or by using a drift net positioned at the major outlets of the sampled springs. Specimens were preserved in 7% formalin solution and dissected in polyvinyl lactophenol. Drawings were made using camera lucida on a Leitz Laborlux phase contrast microscope. Some details gained from scanning electron microscopy (SEM) are added to line drawings. For SEM, 10 females and 8 males of *Phyllognathopus viguieri*, and 4 females and 2 males of *Phyllognathopus bassoti* were dehydrated in a graded ethanol series, critical point dried in a Balzers Union CPD 020 apparatus and coated with gold in a Balzers Union SCD 040 sputter. Observations were made with a Philips SEM XL30 CP scanning electron microscope.

Additional material, preserved on slides, was loaned by the Natural History Museum (London), the Museum National d’Histoire Naturelle (Paris), the National Museum of Natural History, Smithsonian Museum, Washington, D.C. (U.S.A.), the Senckenberg Museum (Germany). The descriptive terminology of Huys and Boxshall (1991) is adopted. Abbreviations used in the text and figures are: P1-P6, first to sixth thoracopods; exp., exopod; enp., endopod; exp (enp) -1 (-2, -3) to denote the proximal (middle, distal) segment of a ramus.

## Results

### Order HARPACTICOIDA Sars, 1903. Family PHYLLOGNATHOPODIDAE Gurney, 1932

#### Genus Phyllognathopus

Mrázek, 1893

http://species-id.net/wiki/Phyllognathopus

##### Emended diagnosis.

Phyllognathopodidae. Habitus slender, with no clear demarcation between prosome and urosome. Integumental dorsal window on cephalosome not confirmed for all members of the genus. Integument with surface pits, moderately sclerotized. Cephalosome rounded; rostrum clearly articulated to cephalosome. First pedigerous somite free. P5-bearing somite with large paired pores laterodorsally. Anal operculum plain or ornamented by fine spinules or extruded in strong spinular processes. Sexual dimorphism in antennule, P5, P6, urosomal segmentation and ornamentation. Female first and second abdominal somites fused forming genital double-somite. Anal somite with paired sensilla on dorsal side. Male urosome consisting of 6 segments. Caudal rami sub-quadrate, or longer than wide, with incomplete setal pattern (6 setae). Dorsal seta inserted on distal third of caudal ramus. Setae III and V variable in morphology among species. Antennule: 8-segmented in female, basically 10-segmented in male, although a suture line marking original segmentation between former segments 10 and 11 may be still discernible in some species; geniculation between segments 7 and 8; segment 9 discrete. Long tube-pores on segments 1 and 2 in both sexes. Antenna: armature of the second endopodal segment consisting of 10 elements. Exopod 1-segmented, with 3 lateral and 2 apical setae. Mandible: mandibular palp biramous, basis unarmed; exopod with 1 apical and 1 inner setae; endopod with 1 inner, 1 subapical and 2 apical setae. Armature of maxillule and maxilla as in *Phyllognathopus viguieri*. Maxilliped: phyllopodial, lamelliform, 1-segmented. Trace of ancestral 2-segmented condition marked by the presence of outer incision; armature consisting of 10 elements.

P1-P3 with praecoxa and 3-segmented exopods and endopods. P4 small-sized, praecoxa missing, with 3- or 2- or 1-segmented exopod and 2- or 1-segmented endopod. Female P5 free, with clear articulation to P5-bearing somite; right and left legs distinct; intercoxal sclerite absent; baseoendopod and exopod coalescent, feeble incision marking original segmentation between them; endopodal lobe not pronounced, bearing 2 apical setae. Exopodal lobe fully incorporated into baseoendopod, not pronounced; basipodal outer seta present. Female P6 present, right and left legs represented by small chitinous lamellar plates, each leg bearing 1 normal seta or a stout spine with rounded tip. Male P5 free, with clear articulation to P5-bearing somite; right and left legs coalescent, intercoxal sclerite absent. Exopod discrete, but sometimes incorporated to basis. Endopod 1-segmented, normally conformed, cylindrical, bearing 1 leaf-like seta, alternatively transformed in a curved and stout element bearing 1 bipinnate seta, inserted on posterior surface of the endopod, close to its articulation to basis. Male P6 present; symmetrical, right and left legs coalescent along their medial margin, forming a continuous lamellar plate; each leg bearing 2 inner spines of different length and 1 outer seta.

#### Phyllognathopus viguieri

(Maupas, 1892)

http://species-id.net/wiki/Phyllognathopus_viguieri

Figs 1 [Fig F2] [Fig F3] [Fig F4] [Fig F5] [Fig F6] [Fig F7] [Fig F8] 9

##### Material examined.

11 ♀♀ and 2 ♂♂,completely dissected and mounted in polyvinyl lactophenol, S. Anna D’Alfaedo, Progno di Valpantena (Verona, Italy), hyporheic habitat, 25.06.2002, E. Gattone coll.; 1 ♀, karstic spring in the hydrogeological basin of Rio Biondo, Progno di Valpantena (Verona, Italy), karstic habitat, 7. 07. 2003, B. Fiasca coll.; 3 ♀♀, Lake Bracciano (Latium, Italy), interstitial habitat, 27.05.02, V. Cottarelli coll.; 10 ♀♀ and 5 ♂♂, Avisio floodplain (Trento, northern Italy), hyporheic habitat, 30.05.2006, T. Di Lorenzo coll.; 3 ♀♀ and 1 ♂, Oignin stream (French Jura Mountains), hyporheic habitat, 30.07.2002, M.-J. Dole-Olivier coll.; 2 ♀♀ and 1 ♂, Ariège floodplain (France), P. Dumas coll.; 1 ♀, Lac Léman (France), slide code MNHN - Cp922, Paris; 2 ♂♂, S. Pierre, (France), slide code MNHN - Cp456, Paris; 3 ♂♂ and 2 ♀♀, Ruhr floodplain (Germany), T. Glatzel coll.; 6 ♀♀ and 1 ♂, R. Krishna, India, Y. Ranga Reddy coll.; 1 ♀, in pitcher of *Nepenthes mirabilis* (Hong Kong), B. Coker det., slide code 1982.329, Natural History Museum, London; 2 ♀♀ deposited at the Smithsonian Institution, Washington D.C., code USMN 251806, 204501; 1 ♂, code USMN 204500; 1 ♂ (juvenile), code USMN 204501.

*Phyllognathopus* cf. *viguieri* A. 2 ♂♂, 2 ♀♀, Madagascar, vial code, MNHN - Cp910, Paris, B. Dussart coll..

*Phyllognathopus* cf. *viguieri* B. 1 ♀, slide code 66/52, freshwater well, Mindoro Island, Philippines, 17.8.1992, V. Cottarelli coll.

*Phyllognathopus viguieri* ?. 10 ♀♀ and 5 ♂♂, Andhra Loyola College Campus, Vijaya-Woda, Andhra Pradesh, India, Y. Ranga Reddy coll..

*Phyllognathopus viguieri menzeli*. 1♀, vial code USNM 150192, labelled: Pacific Ocean, Mariana Islands, Guam, 19 November 1970, Watkins R.L. coll. (remaining material in the vial: 17 ♀♀).

*Phyllognathopus viguieri menzeli* 2. ♂♂, vial code USNM 150193, labelled: Pacific Ocean, Mariana Islands, Guam, 1 April 1971, Belk and Watkins R.L. coll. (remaining material in the vial: 8 specimens, of which several copepodids).

##### Supplementary description.

**Figure 1.**
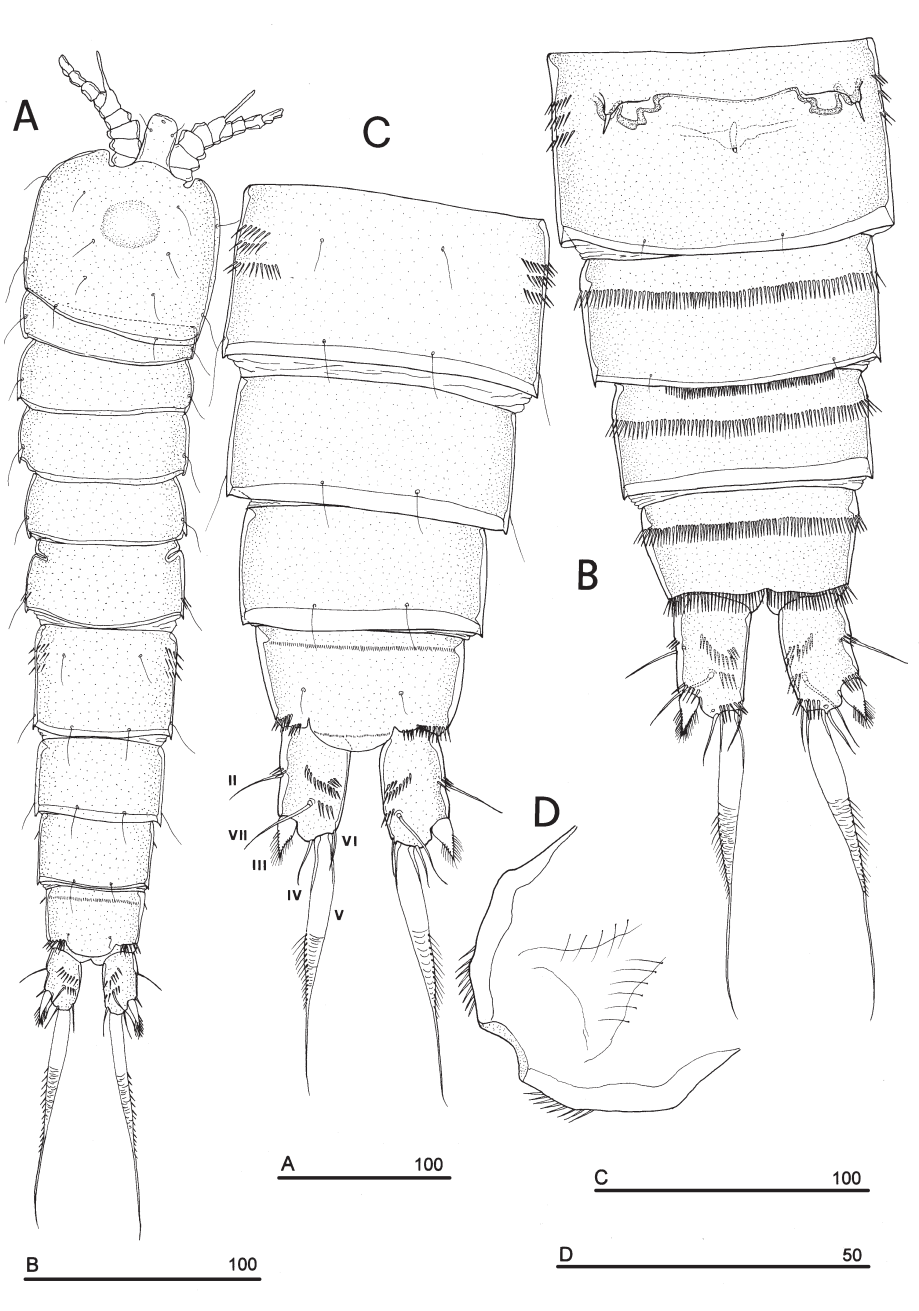
*Phyllognathopus viguieri* (Maupas, 1892) (♀). **A** habitus, dorsal view **B** abdomen, ventral view **C** abdomen, dorsal view **D** labrum (scale bars in μm).

**Figure 2. F2:**
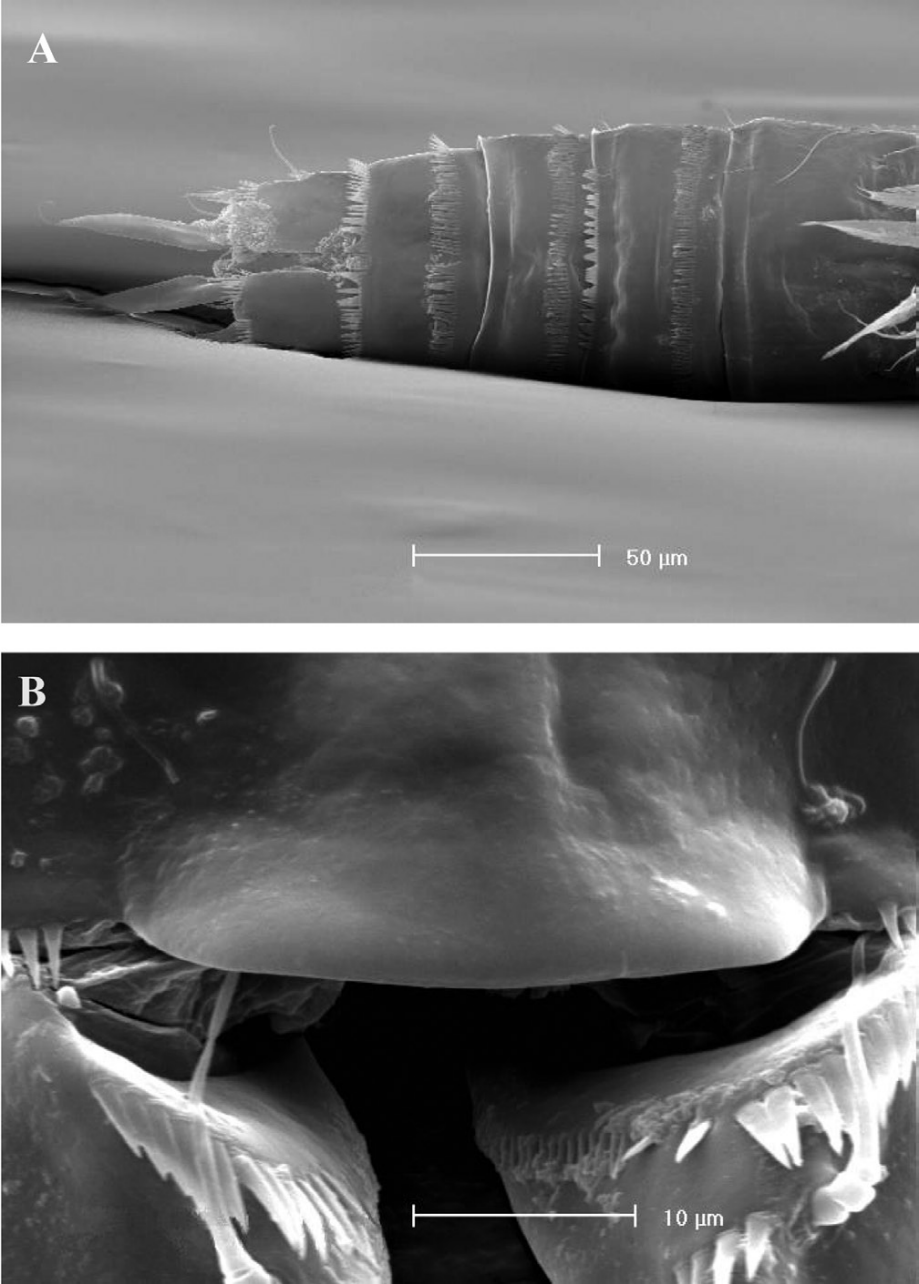
SEM micrographs of *Phyllognathopus viguieri* (Maupas, 1892) (♀). **A** ventral surface of urosome (first urosomite omitted) **B** anal operculum.

**Figure 3. F3:**
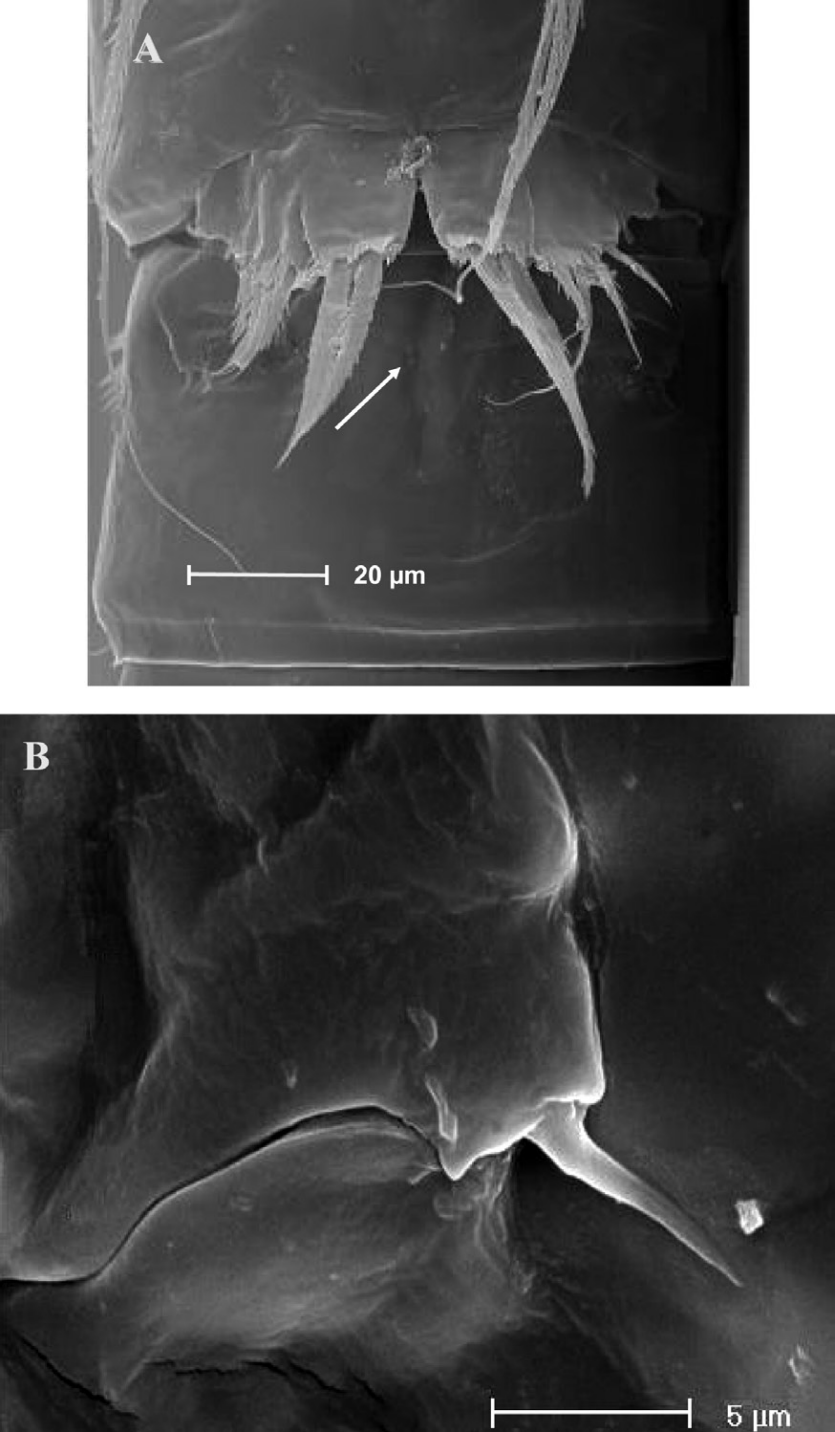
SEM micrographs of *Phyllognathopus viguieri* (Maupas, 1892) (♀). **A** P5 and genital double-somite (copulatory pore arrowed) **B** P6.

FEMALE. Body length, measured from tip of rostrum to posterior margin of caudal rami, from 400 to 600 µm (mean = 439 µm; n = 27). Habitus slender, no clear demarcation between prosome and urosome. Integument with surface pits, moderately sclerotized as in Fig. 1A. Cephalosome sub-quadrate, with a dorsal rounded protuberance, hardly observable, plausibly referable to a dorsal integumental window. Setule pattern as in Fig. 1A. Rostrum elongate, subrectangular in shape, clearly articulated to the cephalosome; two dorsal sensilla laterally inserted on its distal third, and one pore apically. Cephalosome and both thoracic and abdominal somites with cuticular ornamentation apparently represented by reduced number of paired sensilla (Fig. 1A). First pedigerous somite free. Hyaline frills of cephalosome, somites bearing P1-P4 and urosome dorsally smooth. Urosomites with smooth hyaline frill ventrally, except third urosomite (Figs 1B, 2A). Last three urosomites with spinular fringe on proximal third ventrally; anal somite with distal continuous spinule row. Anal somite with paired sensilla on dorsal side only (Fig. 1C), and two short spinule rows close to the anal operculum. Anal operculum rounded, only slightly protruding beyond insertion line of caudal rami (Figs 1C, 2B). P5-bearing somite with large paired pores laterodorsally and paired spinule rows laterally inserted on distal third of somite (Fig. 1A). Genital double-somite with three lateral spinule rows. Female genital field located between first and second third of genital double-somite. Genital apparatus apparently simplified; copulatory pore located halfway of genital double-somite (Figs 1B, 3A). Seminal receptacles located laterally and condensed close to the lamellar P6 (Fig. 3B).

Caudal rami rectangular, parallel, distinctly longer than wide (length/width ratio about 1.7), with incomplete setal pattern (6 setae) (Fig. 1B–C). Anterolateral accessory seta (I) absent, anterolateral seta (II) smooth, inserted at half of caudal ramus; posterolateral seta (III) inserted on distal third of ramus, transformed in a large and stout spiniform element. Outer terminal seta (IV) very short, thin, and naked, shorter than caudal ramus, inner terminal seta (V) unipinnate and relatively short, without articulation at base, with very enlarged proximal part tapering in a subtle tip; terminal accessory seta (VI) slightly shorter than outer terminal seta, thin and naked; dorsal seta (VII) inserted on distal third of caudal ramus, about as long as caudal ramus. Three spinule rows inserted dorsolaterally and two spinule rows inserted at distal margin of caudal ramus ventrally. Two pores located close to the insertion of setae II and IV, ventrally.

**Figure 4. F4:**
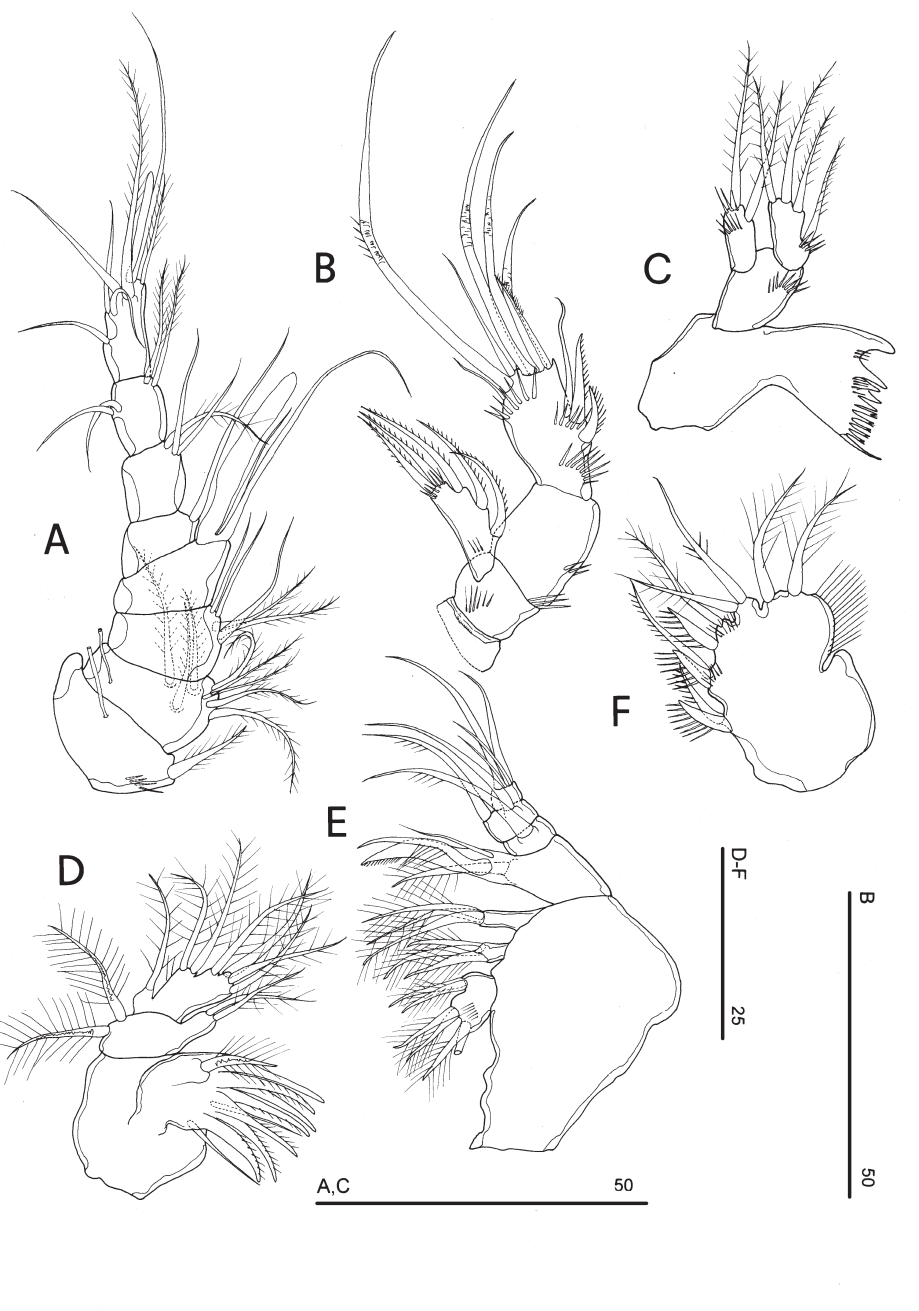
*Phyllognathopus viguieri* (Maupas, 1892) (♀). **A** antennule **B** antenna **C** mandible **D** maxillule **E** maxilla **F** maxilliped (scale bars in μm).

Antennule (Fig. 4A): short, 8-segmented. Segment 1 with 1 spinule row. Both segments 1 and 2 bearing long and flaccid tube-pores. Armature formula: 1-[1], 2-[8], 3-[5], 4-[1 + (1+ae)], 5-[1], 6-[3], 7-[4], 8-[6 + (1+ae)]. Aesthetasc on segment 4 large, reaching about the proximal part of the penultimate antennulary segment.

Antenna (Fig. 4B): coxa unarmed; basis with 1 transverse spinule row on surface, a spinule row inserted on inner margin; exopod 1-segmented, well-defined at base, with surface spinule row, bearing 3 lateral and 2 apical setae; free endopod 2-segmented; both segments robust, of about the same length; segment 1 with inner spinule row; segment 2 with two inner spinule rows; armature consisting of 2 inner spines and 1 seta, 1 unipinnate apical spine, 4 geniculate setae, 1 apical slender seta and 1 subapical slender seta; a row of spinules at outer corner.

Labrum (Fig. 1D): trapezoidal, with two spinule rows on free distal margin. Paired rows of hair-like elements on medioventral surface.

Mandible (Fig. 4C): coxal gnathobase elongate, cutting edge with 2 large and coarse teeth, three smaller teeth and row of fringed teeth; naked seta at dorsal corner. Mandibular palp biramous, basis with inner spinule row, exopod with 1 apical and 1 inner bipinnate setae; endopod with 1 inner, 1 subapical and 2 apical bipinnate setae.

Maxillule (Fig. 4D): well developed arthrite incorporated into praecoxa, with 7 strong curved spines inserted on free distal margin and 1 short seta inserted on a sort of surface peduncle and 2 anterior surface setae. Coxal epipodite represented by 2 setae; coxo-endite with 2 plumose setae. Exopod and endopod incorporated into basis, with a total of 7 plumose setae.

Maxilla (Fig. 4E): syncoxa with 3 endites. Proximal endite with 6 setae; medial and distal endites, each with 3 plumose setae. Allobasis drawn out into a strong claw, distally spinulose, accompanied by 2 robust and 1 thin setae; endopod 3-segmented; segment 1 with 1 robust curved seta; segment 2 with 2 robust curved setae; segment 3 with 2 robust curved and 2 slender setae.

Maxilliped (Fig. 4F): phyllopodial, lamelliform, and 1-segmented. Trace of ancestral 2-segmented condition marked by the presence of outer incision, representing original segmentation boundary between former segments 1 and 2. Armature consisting of 10 elements, of which 5 bipinnate setae in apical position, 1 unipinnate seta inserted along inner margin together with 4 strong unipinnate stout spines. No trace of incision along inner margin.

**Figure 5. F5:**
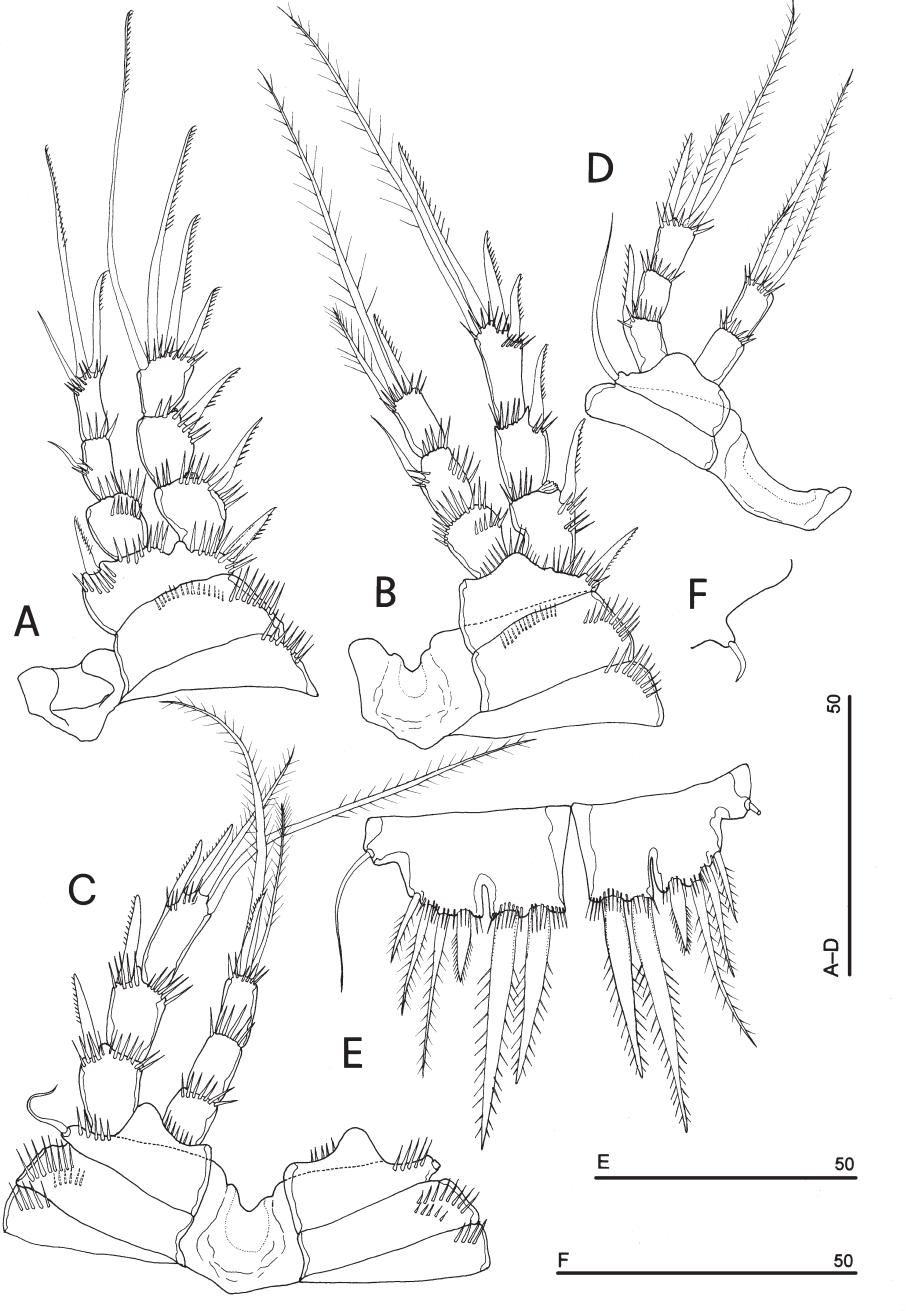
*Phyllognathopus viguieri* (Maupas, 1892) (♀). **A** P1 **B** P2 **C** P3 **D** P4 **E** P5 **F** P6 (scale bars in μm).

P1-P3 with 3-segmented exopods and endopods. P4 with 3-segmented exopod and 2-segmented endopod. Intercoxal sclerites: boundary between intercoxa and basis not well defined at posterior surface of P2-P4 (Fig. 5B–D). P1-P3 praecoxa well developed, with outer spinule row. P4 praecoxa absent.

P1 (Fig. 5A): praecoxa and coxa with outer spinule row on anterior surface; one posterior row of thin spinules inserted close to coxo-basis boundary. Basis with 1 outer spiniform seta and 1 inner spine, with spinule rows along outer margin, between exopod and endopod and at the insertion of inner spine, respectively. Exopod about as long as endopod; exp-1 and -2 with 1 outer unipinnate spine; exp-3 with 2 unipinnate spines in apical position, and 1 apical and 1 subapical inner setae. Endopod: enp-1 unarmed, about as long as enp-2 and enp-3, wider than enp-2 and enp-3. Enp-2 cylindrical, with short inner seta inserted at the middle of segment. Enp-3 with 1 inner spine, 1 apical seta and 1 curved apical spine. Ornamentation as in Fig. 5A.

P2 (Fig. 5B): ornamentation of praecoxa and coxa as in P1. Basis with 1 outer spine, with spinule rows along outer margin, and between exopod and endopod. Exopod slightly longer than endopod; exopodal segments of about the same length; exp-1 and -2 with 1 outer unipinnate spine; exp-3 with 2 outer unipinnate spines, 1 apical unipinnate seta and 1 subapical long inner seta. Endopod: enp-1 unarmed; enp-2 with 1 naked inner seta; enp-3 with 1 spine and 1 long bipinnate seta in apical position, and 1 short bipinnate subapical seta. Ornamentation as in Fig. 5B.

P3 (Fig. 5C): ornamentation of praecoxa and coxa as in P2. Basis with short outer seta and spinule rows along outer margin and at the insertion of the endopod. Exopod distinctly longer than endopod. Exp-1 and -2 with 1 unipinnate outer spine; exp-3 with 2 unipinnate outer spines, 1 bipinnate apical seta and 1 long bipinnate subapical seta. Endopod: enp-1 and -2 unarmed; enp-3 with 1 spine and 2 bipinnate setae in apical position. Ornamentation as in Fig. 5C.

P4 (Fig. 5D): reduced in size, praecoxa absent, coxa and basis without ornamentation; basis with long outer naked seta; exopod and endopod about as long as half of remaining legs; the exopod only slightly longer than endopod. Exp-1 with 1 unipinnate outer spine; exp-2 unarmed; exp-3 with 1 bipinnate outer spine and 2 apical setae of different length. Endopod: enp-1 unarmed; enp-2 with 3 apical setae. Ornamentation as in Fig. 5D.

P5 (Figs 3A, 5E): free, with clear articulation to P5-bearing somite; right and left legs distinct; baseoendopod and exopod coalescent, incision marked original segmentation between them still present; basipodal outer seta present, exopodal armature consisting of 3 bipinnate setae and 1 stout spine: all elements in apical position; baseoendopod armed with 2 robust bipinnate setae, the outermost the longest.

P6 (Figs 3B, 5F): rudimentary, consisting of paired small chitinous lamellar plates not coalescent along medial margin, partially covering seminal receptacles. Armature consisting of 1 short naked spine with rounded tip on each leg.

##### Male.

**Figure 6. F6:**
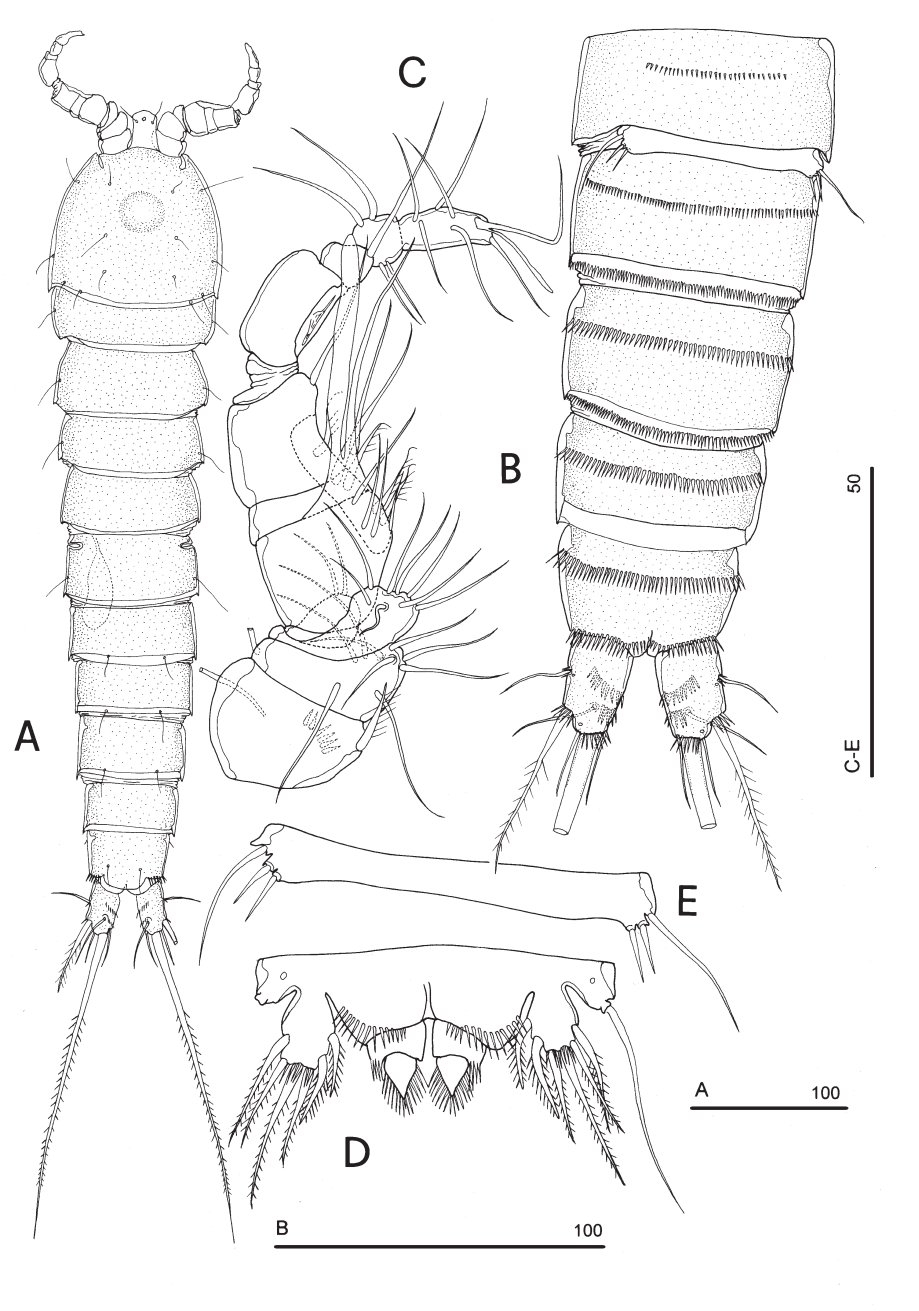
*Phyllognathopus viguieri* (Maupas, 1892) (♂). **A** habitus, dorsal view **B** abdomen, ventral view (first urosomite omitted) **C** antennule **D** P5 **E** P6 (scale bars in μm).

**Figure 7. F7:**
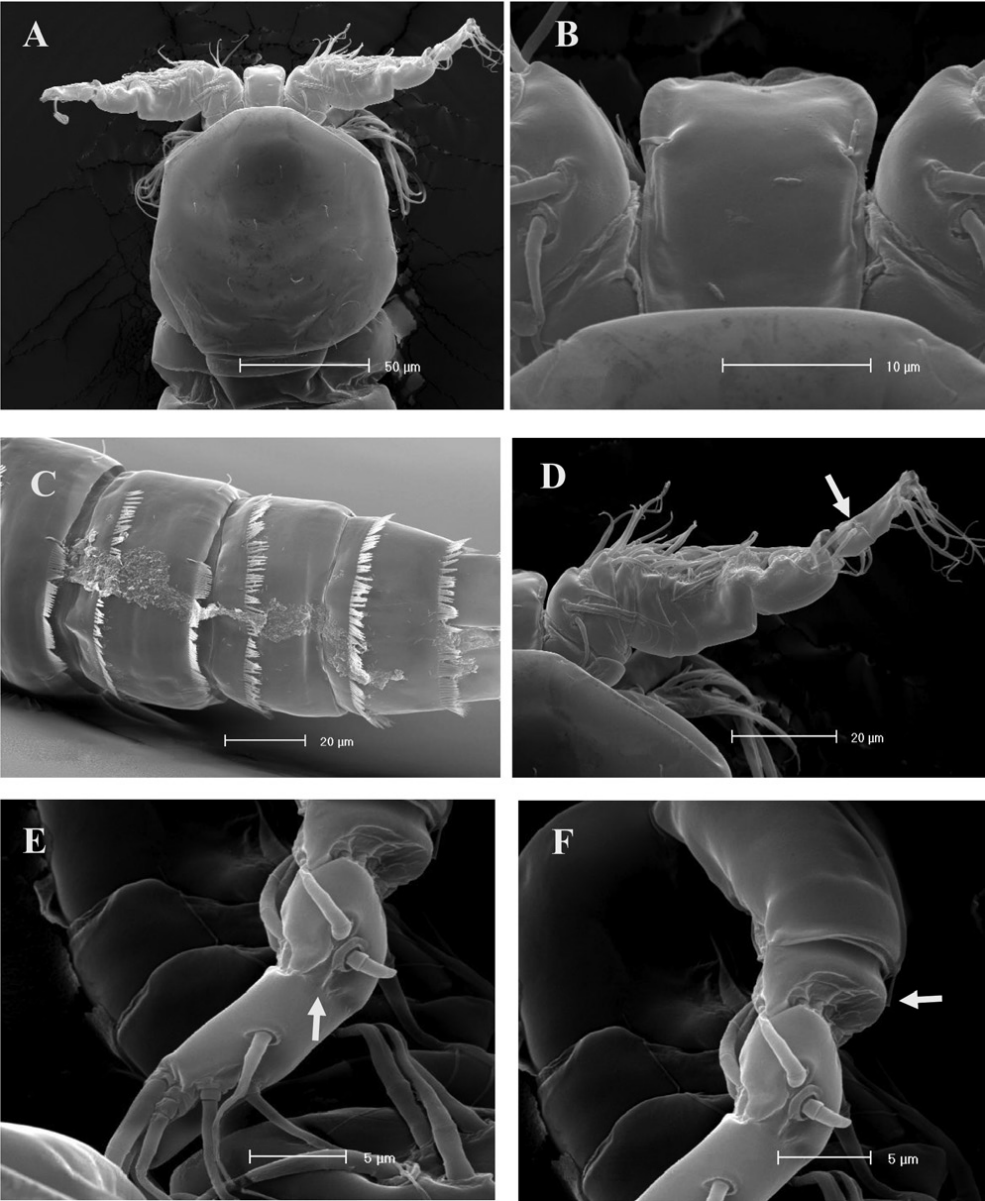
SEM micrographs of *Phyllognathopus viguieri* (Maupas, 1892) (♂). **A** cephalosome, rostrum and antennule **B** rostrum (detail) **C** ornamentation of third to six urosomites, ventral surface **D** antennule, segments 10 and 11 discrete on posterior surface (boundary line arrowed) **E** antennule, detail of segments 10 and 11 fused on anterior surface (suture line arrowed) **F** antennule, segment 9 arrowed.

Body length, measured from tip of rostrum to posterior margin of caudal rami, from 370 to 541 µm, with mean of 424 µm based on 8 individuals. Sexual dimorphism in antennule, abdominal segmentation, P5, P6 and caudal setae morphology. Habitus, cephalosome (Figs 6A, 7A–B), sensilla and pore patterns as in female. Integument with surface pits. Urosome as in Figs 6B, 7C. Caudal rami with 6 setae (Fig. 6B). Anterolateral seta (II) as in female, posterolateral seta (III) setiform, not transformed (length seta/length caudal ramus: about 2) and bipinnate. Outer terminal seta (IV) as in female, inner terminal seta (V) not transformed, plumose and long, not articulated at base; terminal accessory seta (VI) and dorsal seta (VII) as in female (Fig. 6). Ornamentation and pore patterns as in female. Anal operculum as in female.

**Figure 8. F8:**
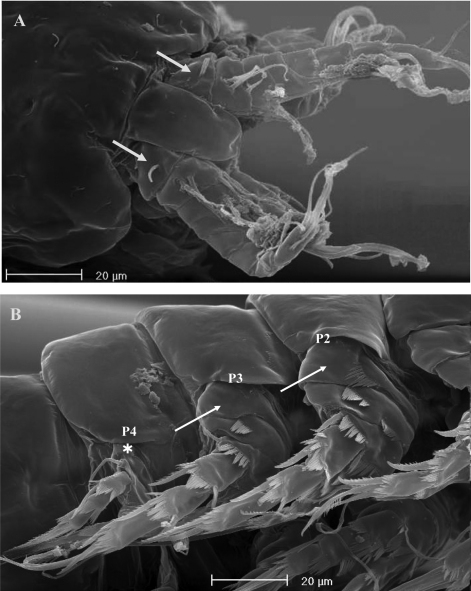
SEM micrographs of *Phyllognathopus viguieri* (Maupas, 1892) (♂). **A** antennule, tube-pores on the first antennulary segment arrowed **B** P2-P4 (P2-P3 praecoxa arrowed; asterisk indicates the P4 praecoxa missing).

Antennule (Figs 6C, 7D–F): elongate, basically 10-segmented, last segment still showing a surface suture line only on anterior surface, indicating an incipient 11-segmented condition. Segment 1 with 1 ventral spinule row and 1 tube-pore (Fig. 8A). Segment 2 with tube-pore. Segment 4 represented by small U-shaped sclerite. Segment 6 the largest, sclerotized. Segment 8 elongate and transformed, moderately sclerotized, segment 9 distinct, not incorporated into segment 8, segment 10 derived by incomplete fusion between former segments 10 and 11 (Fig. 7D–F). Armature formula: 1-[1], 2-[9], 3-[8], 4-[2], 5-[7+(1 + ae)], 6-[2], 7-[2], 8-[0], 9-[1], 10-[10 + (1 + ae)]. Aesthetasc on segment 5 very large. Segment 8 with medial pointed protrusions as in Fig. 6C.

**Figure 9.**
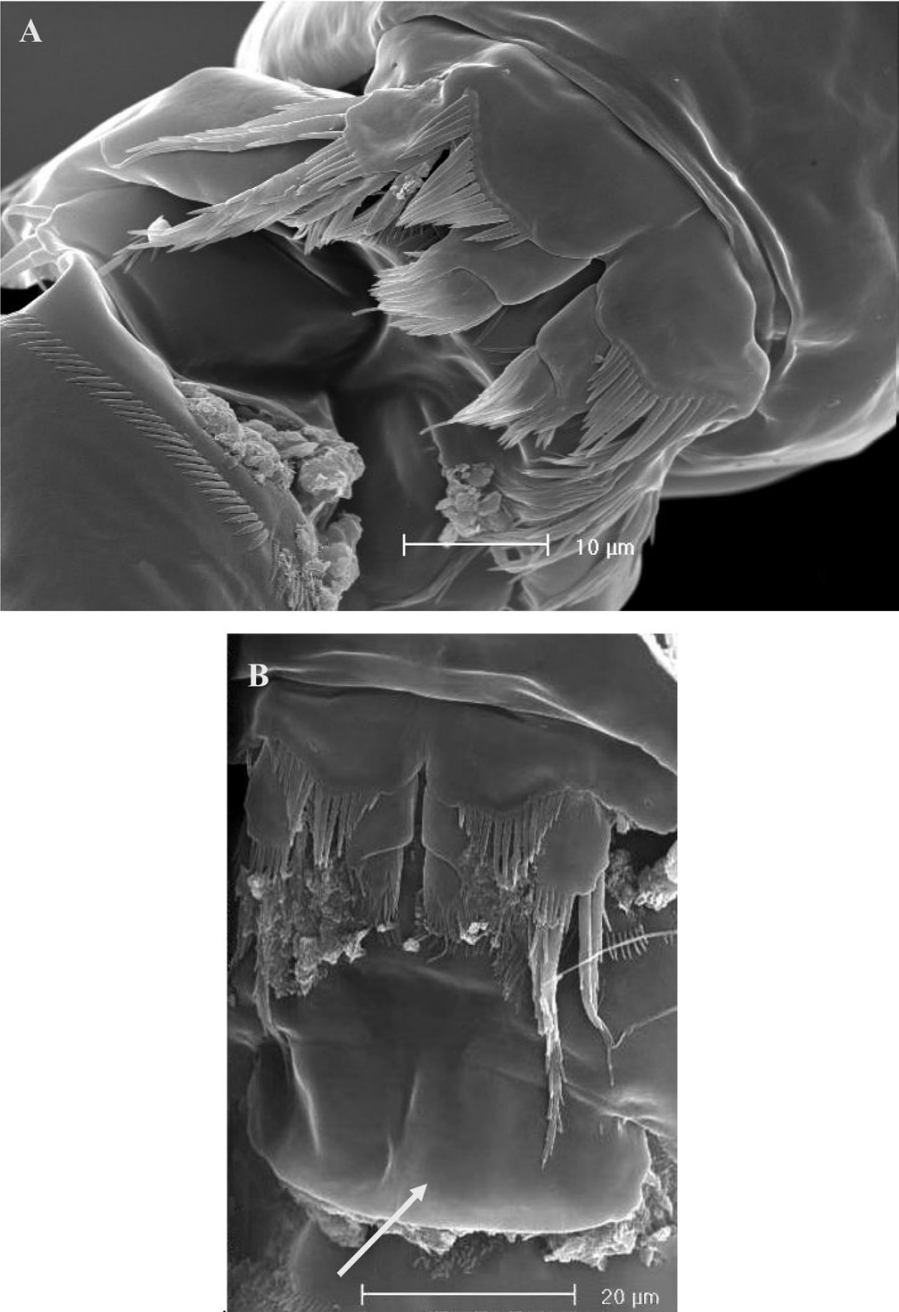
SEM micrographs of *Phyllognathopus viguieri* (Maupas, 1892) (♂). **A** P5 **B** P6 (arrowed).

P1-P4 as in female; for morphological details of P1-P4 see Fig. 8B. P5 (Figs 6D, 9A): free, with clear articulation to P5-bearing somite; right and left legs coalescent; exopod clearly discernible but incorporated to basis: no trace of articulation between them observable, bearing 2 inner, 2 apical and 2 outer bipinnate setae; endopod discrete, distinctly 1-segmented, bearing 1 large leaf-like transformed seta and a spinule row along its free outer margin. Basipodal outer seta slender and naked, one pore near its insertion.

P6 (Figs 6E, 9B): right and left legs coalescent, forming a single linear lamellar plate, with no trace of incision between right and left P6; armature consisting of 2 spines and 1 outer seta.

#### Phyllognathopus inexspectatus

Galassi & De Laurentiis sp. n.

urn:lsid:zoobank.org:act:0BCBB7D6-0796-49AE-8856-BE37771B0F66

http://species-id.net/wiki/Phyllognathopus_inexspectatus

**Figure 10.**
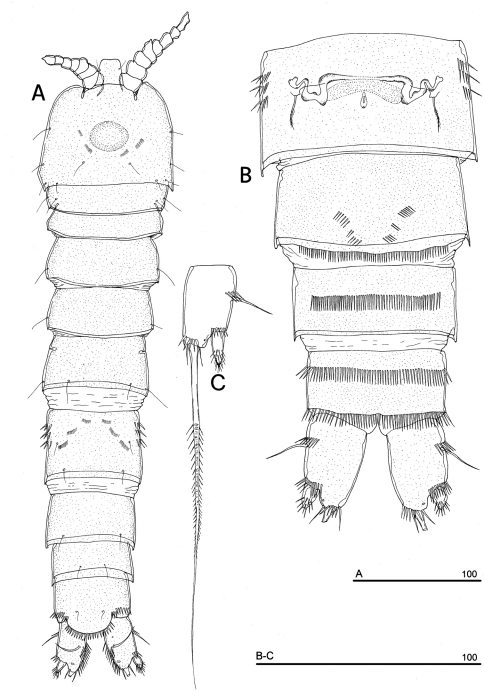
*Phyllognathopus inexspectatus* sp. n. (♀). **A** habitus, dorsal view **B** abdomen, ventral view **C** caudal ramus, ventral view (scale bars in μm).

Figs 10 [Fig F11] [Fig F12] 13

Phyllognathopus sp. (in Di Lorenzo et al. 2005). (Synonymy)

##### Type material.

♀ holotype completely dissected and mounted in glycerine, deposited at the Natural History Museum, London (UK); January 2003, D. Cipriani coll.; 3 ♀♀ paratypes completely dissected and mounted in lactophenol; May 2003; January 2004. D. Cipriani coll..

##### Type locality.

Mazzoccolo karstic spring (Latium, central Italy), coordinates: 41°15'17"N, 13°27'08"E; Western Aurunci Mountains; 20 m a.s.l.; water temperature 13.5 ± 0.3 °C; pH 7.5 ± 0.1; O2 9.1 ± 0.9 mg/L (n = 11).

##### Description.

FEMALE. Total body length, measured from tip of rostrum to posterior margin of caudal rami, 474 µm (holotype), 468 µm (paratypes mean value; n = 3). Body depigmented and eyeless. Habitus slender, no clear demarcation between prosome and urosome. Integument with surface pits, moderately sclerotized. Cephalosome subquadrate, with a dorsal rounded protuberance, hardly observable, referable to the dorsal integumental window (Fig. 10A). Couples of setule rows located on surface of cephalic shield. Rostrum elongate, subrectangular in shape, clearly articulated to the cephalosome; two dorsal sensilla laterally inserted on distal third, and one pore apically. Cephalosome and both thoracic and abdominal somites with cuticular ornamentation represented by reduced number of paired sensilla (Fig. 10A). First pedigerous somite free. Hyaline frills of cephalosome, somites bearing P1-P4 and urosome dorsally smooth. P5-bearing somite with large paired pores laterodorsally. Genital double-somite with three lateral spinule rows and three pairs of setule rows inserted dorsally. Female genital field located at the middle of genital double-somite. Genital apparatus simplified, copulatory pore located at half of the genital double-somite. Seminal receptacles laterally located and condensed close to the lamellar sixth legs.

Urosomites with smooth hyaline frill ventrally, except third urosomite (Fig. 10B). Last two urosomites with spinular fringe on proximal third; anal somite with distal continuous spinule row.

**Figure 11. F11:**
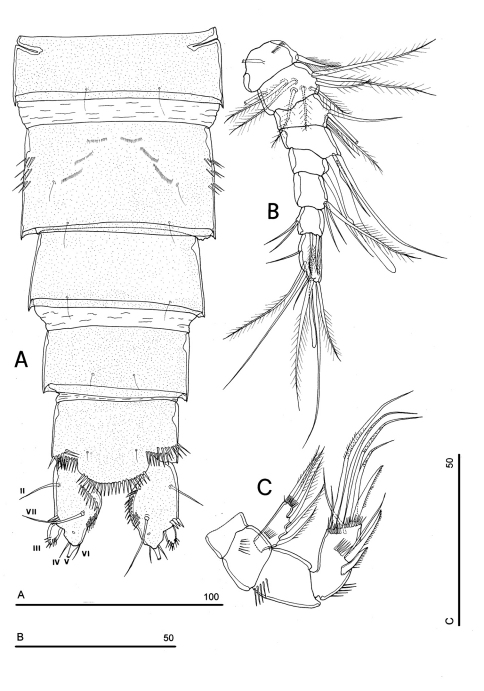
*Phyllognathopus inexspectatus* sp. n. (♀) **A** urosome, dorsal view **B** antennule **C** antenna (scale bars in μm).

Anal somite with paired sensilla on dorsal surface (Fig. 11A), and two short spinule rows close to the anal operculum. Anal operculum rounded, protruding beyond insertion line of caudal rami and armed with strong spinules on free distal margin (Fig. 11A). Caudal rami rectangular with strongly expanded inner margin, slightly divergent, distinctly longer than wide (length/width ratio: about 1.5), with incomplete setal pattern (6 setae) (Figs 10C, 11A); anterolateral accessory seta (I) absent, anterolateral seta (II) smooth, inserted at proximal third of caudal ramus; posterolateral seta (III) inserted on distal third of ramus, transformed in a short and stout spiniform seta, with tuft of spinules apically. Outer terminal seta (IV) very short, thin, and naked, without articulation at base (Fig. 10C), distinctly shorter than caudal ramus; inner terminal seta (V) not transformed, very long, without articulation at base; terminal accessory seta (VI) as long as outer terminal seta, thin and naked; dorsal seta (VII) inserted at half of caudal ramus, about as long as caudal ramus or slightly shorter. A continuous spinule row along inner margin of caudal ramus and three spinule rows inserted close to the anterolateral seta (Figs 10B, 11A), at the basis of the posterolateral seta and at distal margin of ramus ventrally, respectively. Two pores are located dorsally on each caudal ramus, and one pore ventrally.

Antennule (Fig. 11B): short, 8-segmented. Segment 1 with ventral spinule row. Both segments 1 and 2 bearing long and flaccid tube-pores. Armature formula: 1-[1], 2-[8], 3-[5], 4-[1 + (1 + ae)], 5-[1], 6-[3], 7-[4], 8-[6 +(1+ ae)]. Aesthetasc on segment 4 very large, reaching about the last antennulary segment.

Antenna (Fig. 11C): coxa unarmed; basis with 1 transverse spinule row on surface; exopod 1-segmented, well-defined at base, with spinule row on surface, bearing 3 lateral unipinnate and 2 apical bipinnate setae; free endopod 2-segmented; both segments robust, of about the same length; segment 1 with inner spinule row; segment 2 with one inner and one surface spinule rows; armature consisting of 2 inner spines and 1 thin seta, 1 apical unipinnate spine, 4 geniculate setae, and 1 apical and 1 surface slender setae; two rows of spinules at outer corner and in subapical position on free distal margin, respectively.

**Figure 12. F12:**
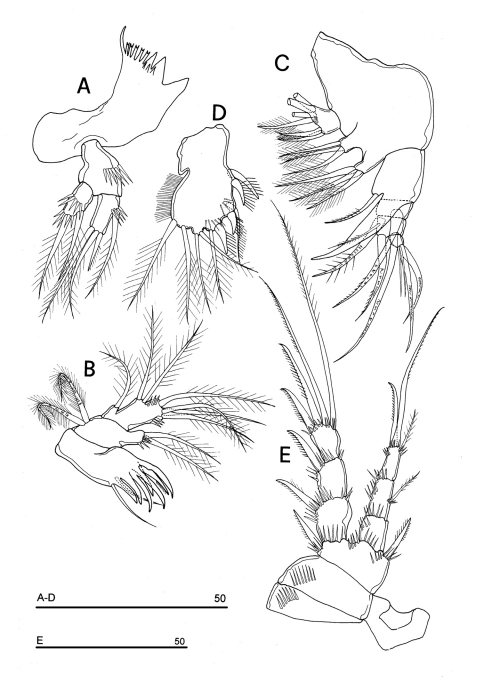
*Phyllognathopus inexspectatus* sp. n. (♀). **A** mandible **B** maxillule **C** maxilla **D** maxilliped **E** P1 (scale bars in μm).

Mandible (Fig. 12A): coxal gnathobase elongate, cutting edge with 3 large and coarse teeth, 5 smaller fringed teeth; naked seta at dorsal corner. Mandibular palp biramous, basis with inner strong spinule row, exopod with 1 apical and 1 inner bipinnate setae; endopod with 1 inner bipinnate, and 1 spiniform and 2 bipinnate apical setae. Ornamentation as in Fig. 12A.

Maxillule (Fig. 12B): well developed arthrite incorporated into praecoxa, with 7 strong curved spines inserted on free distal margin, and 2 anterior surface setae. Proximal surface bipinnate seta inserted on tubercle absent (vs. present in *Phyllognathopus viguieri*, see Fig. 4D). Coxal epipodite represented by 2 setae; coxo-endite with 2 plumose setae. Exopod and endopod incorporated into basis, bearing 7 plumose setae.

Maxilla (Fig. 12C): syncoxa with 3 endites. Proximal endite free, with 6 setae; medial and distal endites incorporated to syncoxa, each with 3 plumose setae, inserted as in Fig. 12C. Allobasis drawn out into a strong claw apparently smooth, accompanied by 1 robust and 2 thin setae; endopod 3-segmented; segment 1 with 1 robust curved seta; segment 2 with 2 robust curved setae; segment 3 with 2 robust curved and 2 slender setae.

Maxilliped (Fig. 12D): phyllopodial, lamelliform, 1-segmented, and slender than in *Phyllognathopus viguieri*. Trace of ancestral 2-segmented condition marked by the presence of outer incision, probably representing original segmentation boundary between segments 1 and 2. Armature consisting of 10 elements, of which 5 bipinnate setae in apical position, two of which with independent insertion, 1 unipinnate seta inserted along inner margin together with 4 strong unipinnate spines. No trace of incision along inner margin.

P1-P3 with 3-segmented exopods and endopods. P4 with 2-segmented exopod and endopod. Intercoxal sclerites: boundary between intercoxa and basis not well defined at posterior surface in P2-P4. P1-P3 praecoxa well developed, with 1 outer spinule row. P4 praecoxa absent.

P1 (Fig. 12E): praecoxa and coxa with outer spinule row on anterior surface. Basis with 1 outer spiniform seta and 1 inner spine; with spinule rows along outer margin, between exopod and endopod and at the insertion of inner spine, respectively. Exopod slightly longer than endopod: exp-1 and -2 with 1 outer unipinnate spine; exp-3 with 2 outer unipinnate spines and 2 setae, respectively inserted apically and subapically. Endopod: enp-1 unarmed, about as long as enp-2 and enp-3. Enp-2 cylindrical, with 1 inner short seta. Enp-3 with 1 inner bipinnate seta, 1 long unipinnate curved seta and 1 spiniform curved seta in apical position. Ornamentation as in Fig. 12E.

**Figure 13.**
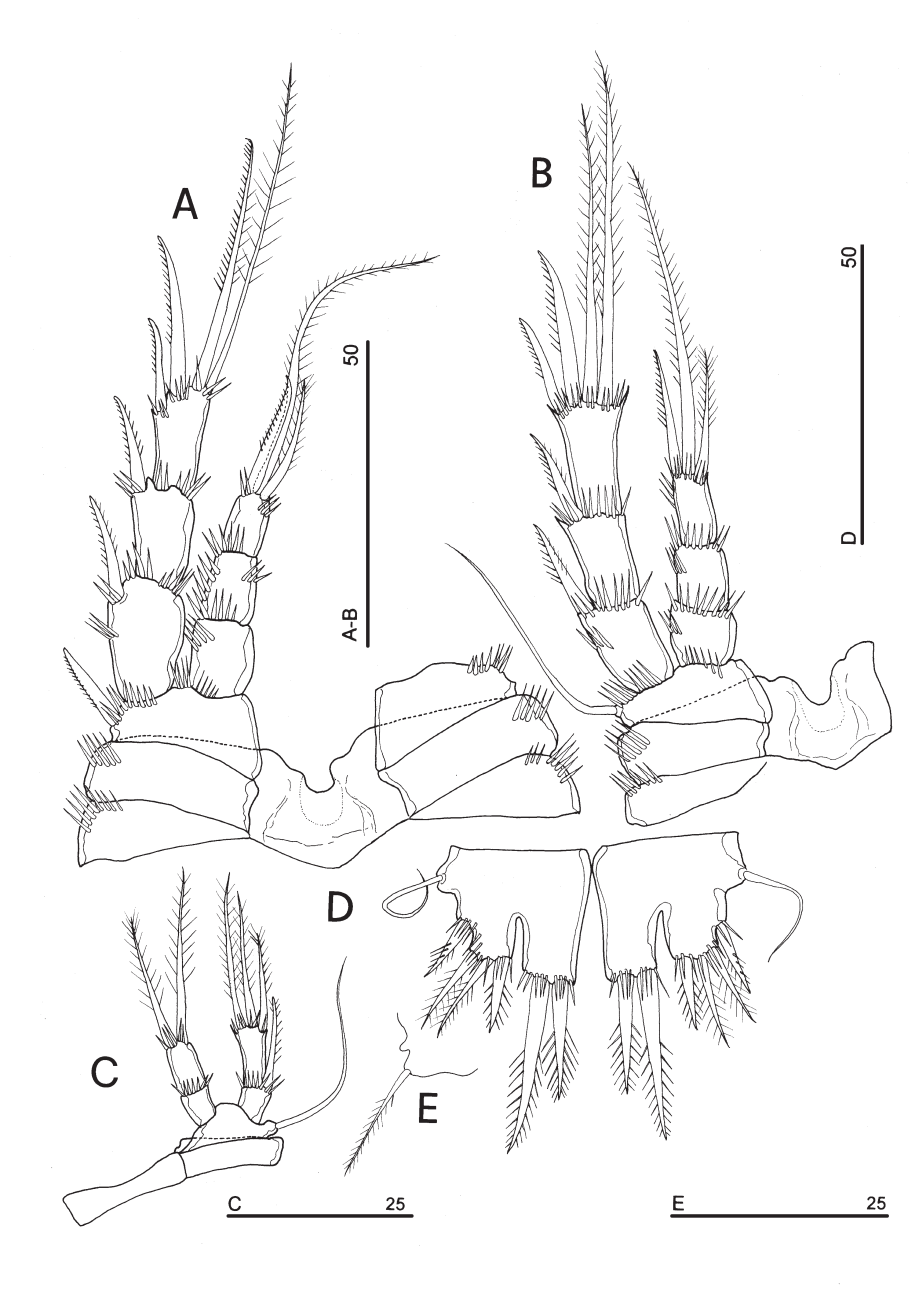
*Phyllognathopus inexspectatus* sp. n. (♀). **A** P2 **B** P3 **C** P4 **D** P5 **E** P6 (scale bars in μm).

P2 (Fig. 13A): praecoxa and coxa as in P1; basis with 1 outer spiniform seta, with spinule rows along outer margin, and between exopod and endopod. Exopod distinctly longer than endopod; exopodal segments of about the same length; exp-1 and -2 with 1 outer bipinnate spine; exp-3 with 2 outer unipinnate spines, 1 apical unipinnate seta and 1 subapical long bipinnate seta. Endopod: enp-1 and-2 unarmed; enp-3 with 1 spine and 1 long bipinnate seta in apical position, 1 subapical short bipinnate seta. Ornamentation as in Fig. 13A.

P3 (Fig. 13B): ornamentation of praecoxa and coxa as in P1-P2. Basis with long outer seta and spinule rows along outer margin and at the insertion of the endopod. Exopod distinctly longer than endopod. Exp-1 and -2 with 1 outer bipinnate spine; exp-3 with 2 outer unipinnate spines, and 2 apical long bipinnate setae. Endopod: enp-1 and -2 unarmed; enp-3 with 1 unipinnate spine and 1 long bipinnate seta in apical position, 1 subapical bipinnate seta. Ornamentation as in Fig. 13B.

P4 (Fig. 13C): small sized, if compared to P1-P3; praecoxa absent, coxa and basis without ornamentation; basis with outer long and naked seta; exopod as long as endopod. Exp-1 with 1 outer long unipinnate spine; exp-2 with 1 outer long spine and 2 apical setae. Endopod: enp-1 unarmed; enp-2 with 2 apical plumose setae. Ornamentation as in Fig. 13C.

P5 (Fig. 13D): free, with clear articulation to P5-bearing somite; right and left legs distinct; baseoendopod and exopod coalescent, incision marked original segmentation still present; basipodal outer seta present, exopodal armature consisting of 1 outer spine, 2 apical short setae, of about the same length, and 1 apical spine; baseoendopod armed with 2 robust bipinnate setae, the outer the longest.

P6 (Fig. 13E): rudimentary, consisting of small paired chitinous lamellar plates not coalescent along medial margin, partially covering the genital field. Armature consisting of 1 long and slender bipinnate seta on each side.

Male unknown.

##### Etymology.

The specific name derives from the Latin adjective *inexspectatus* which means “unexpected”, alluding to the surprising geographical location of the species, being the taxonomically related *Phyllognathopus* distributed in the Southern Hemisphere, and to the ecological finding of this species, which was collected from a large karstic aquifer in Central Italy, whereas all the other members of the genus are epigean.

##### Ecology.

At present knowledge the species is to be considered a stygobiotic species, collected from a karstic aquifer of the Western Aurunci Mountains (Latium) (Di Lorenzo et al. 2005). Although this aquifer is intensively fissured and karstified, with diffuse landforms of sinkholes and a discharge which is strictly linked to rainy events, stygoxene species were only sporadically present and represented by few individuals, due to the absence of a surface hydrological network, a landscape feature which is typical for coastal Mediterranean areas.

### Genus Parbatocamptus Dumont and Maas, 1988

#### Parbatocamptus jochenmartensi

Dumont and Maas, 1988

http://species-id.net/wiki/Parbatocamptus_jochenmartensi

Figs 14–16

##### Material examined.

♂ holotype, completely dissected and mounted on slide labeled SMF 14657, deposited at the Senckenberg Museum (Germany).

##### Emended diagnosis of the genus Parbatocamptus Dumont and Maas, 1988.

Phyllognathopodidae. Body flattened. First pedigerous somite free. Hyaline frills of abdominal somites ventrally smooth. Anal operculum prominent, subdistally crenulated. Caudal ramus subquadrate in ventral view, tapering on free distal margin in dorsal view, with 6 setae; anterolateral accessory seta (I) absent; dorsal seta inserted on spinulose protuberance closely located to free distal margin. Male antennule 10-segmented, with geniculation between segments 7 and 8; segment 9 distinct, as usual in the family; tube-pores on segments 1 and 2; aesthetascs on segments 5 and 10. Antenna: exopod 1-segmented, well-defined at base, bearing 3 lateral and 2 apical unipinnate setae; free endopod 2-segmented; both segments robust, of about the same length. Mandibular palp biramous; basis with inner long bipinnate seta; exopod with 2 setae; endopod with 2 apical geniculate setae; 1 inner and 1 subapical bipinnate setae. Maxillary syncoxa with 3 endites. Proximal endite quadrilobate, with 6 apical setae; medial and distal endites, each with 3 setae. Allobasis drawn out into a strong claw, accompanied by 1 curved and 2 normal setae; endopod 3-segmented; segment 1 with 1 robust curved seta; segment 2 with 2 robust curved setae; segment 3 with 2 robust curved and 2 slender setae. Maxilliped phyllopodial, lamelliform, 1-segmented. Clear trace of ancestral 2-segmented condition well discernible and marked by outer and inner incisions; armature consisting of 11 elements: 1 strong spine inserted on inner corner of former proximal segment; 4 spines and 6 setae along free distal margin of former second segment, two of which inserted on independent little knob. P1-P2 with 3-segmented exopods and endopods. P3-P4 with 3-segmented exopods and 2-segmented endopods. P1-P4 praecoxa present. P1 exp-1 long, about as long as exp-2 and -3 together. P1-P4 endopods distinctly shorter than exopods, not overreaching distal margin of exp-2. P2 enp-2 transformed: outer margin produced into a comb-like structure; P2 enp-3 with 1 apical strong curved spine and 1 apical seta. P5 with 2-segmented exopod; endopod incorporated to basis, forming a baseoendopod; suture line still observable on posterior surface; rudimentary intercoxal sclerite still discernible. P6 symmetrical, consisting of a well developed, deeply incised lamella, marking original division between left and right legs. Armature consisting of 1 outer seta and 2 inner short spines of different length.

Female unknown.

##### Supplementary description of the holotype.

The description deals with major morphological details, omitted or overlooked in the original description, and with improvements of observational errors.

Integumental pitting not detectable on the dissected holotype; integument well sclerotized.

**Figure 14.**
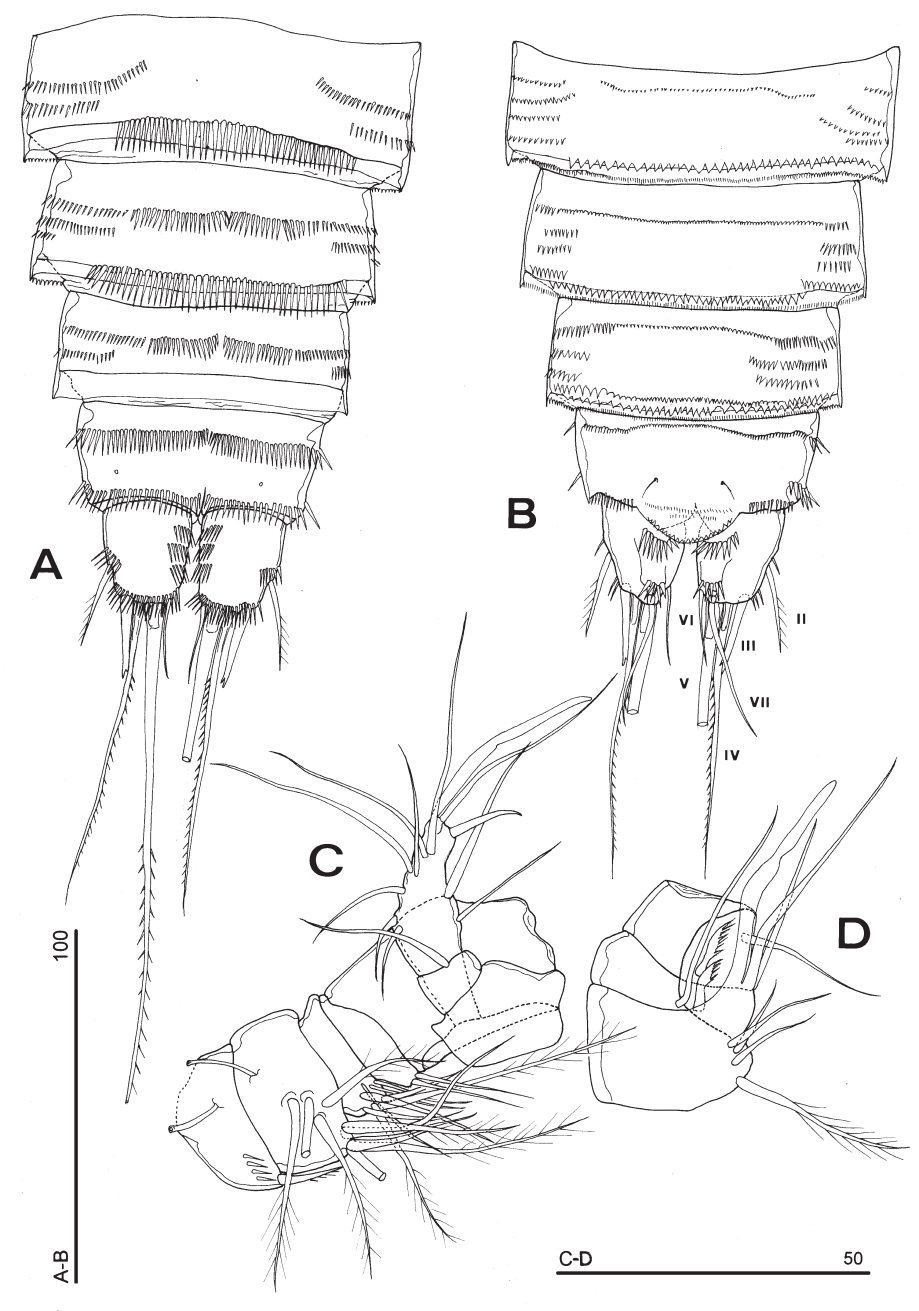
*Parbatocamptus jochenmartensi* Dumont and Maas, 1988 (♂). **A** abdomen, ventral view **B** abdomen, dorsal view **C** antennule **D** antennule, detail of segments 5-7 (scale bars in μm).

Urosomites 3–5 with smooth hyaline frills ventrally (Fig. 14A); third and fourth urosomites with spinular fringe closely located to hyaline frill; fifth urosomite with surface spinule rows ventrally, anal somite with distal continuous spinule row. Other ornamentation as in Fig. 14A. Urosomites 3–5 with crenulated hyaline frills dorsally. Indented surface rows are observable on third, fourth and fifth urosomites dorsally (Fig. 14B). Anal somite with row of fine spinules on proximal third; spinule row at the insertion of each caudal ramus; paired dorsal sensilla. Anal operculum slightly protruding the insertion line of caudal rami, subdistally crenulated (Fig. 14B).

Caudal rami ventrally subquadrate, parallel, longer than wide (length/width ratio: about 1.4), with incomplete setal pattern (6 setae) (Fig. 14A–B). Anterolateral accessory seta (I) absent, anterolateral seta (II) unipinnate, inserted at second third of caudal ramus; posterolateral seta (III) inserted in subdistal position, transformed in spiniform bifid seta. Outer terminal seta (IV) well developed, unipinnate, with articulation at base; inner terminal seta (V) bipinnate and long; terminal accessory seta (VI) thin and naked, about as long as posterolateral seta; dorsal seta (VII) inserted on a spinulose knob, close to free distal margin of caudal ramus, distinctly longer than caudal ramus. A spinule row on proximal third of each caudal ramus dorsally; three spinule rows inserted ventrolaterally and one spinule row at distal margin of caudal ramus, ventrally.

Antennule (Fig. 14C): 10-segmented. Segment 1 with spinule row and tube-pore. Segment 2 with tube-pore. Segment 4 represented by small U-shaped sclerite. Segment 5 large, sclerotized (Fig. 14D). Segment 8 very large and transformed, moderately sclerotized, segment 9 short, discrete, segment 10 derived by complete fusion of former segments 10 and 11. Armature formula: 1-[1], 2-[9], 3-[8], 4-[2], 5-[5?+(1 + ae)], 6-[2], 7-[2], 8-[0], 9-[1], 10-[10 + (1 + ae)].

**Figure 15.**
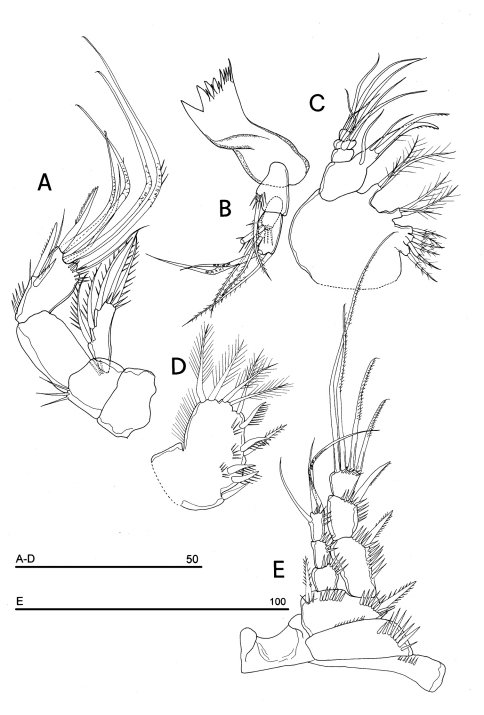
*Parbatocamptus jochenmartensi* Dumont and Maas, 1988 (♂). **A** antenna **B** mandible **C** maxilla **D** maxilliped **E** P1 (scale bars in μm).

Antenna (Fig. 15A): coxa unarmed; basis with 1 transverse spinule row on surface, a spinule row inserted on apical inner margin; exopod 1-segmented, well-defined at base, bearing 3 lateral and 2 apical unipinnate setae; free endopod 2-segmented; both segments robust, of about the same length; segment 1 naked; segment 2 with one inner spinule row, armature consisting of 2 inner spines and 1 seta, 1 apical unipinnate spine, 4 geniculate setae, 1 apical slender seta and 1 subapical tiny seta; a row of spinules at outer corner.

Mandible (Fig. 15B): coxal gnathobase elongate, cutting edge with 2 large and coarse teeth, three smaller teeth and row of tiny teeth; naked seta at dorsal corner. Mandibular palp biramous, basis with inner long bipinnate seta and spinule row, exopod with 2 bipinnate setae; endopod with 2 apical geniculate setae, 1 inner and 1 subapical bipinnate setae.

Maxillule not observable.

Maxilla (Fig. 15C): syncoxa with 3 endites fully incorporated to syncoxa. Proximal endite quadrilobate, with 6 setae; the first two distal lobes each bearing 2 setae; the proximal ones, each with 1 plumose seta; medial and distal endites, each with 3 bipinnate setae. Allobasis drawn out into a strong claw, distally spinulose, accompanied by 1 robust and curved seta and 2 naked setae, respectively; endopod 3-segmented; segment 1 with 1 robust curved seta; segment 2 with 2 robust curved setae; segment 3 with 2 curved and 2 slender setae.

Maxilliped (Fig. 15D): phyllopodial, lamelliform, 1-segmented. Clear trace of ancestral 2-segmented condition marked by the presence of both outer and inner incisions. Armature consisting of 11 elements: 1 strong spine inserted at inner corner of former segment 1; 4 strong unipinnate spines along inner margin, 1 bipinnate seta inserted along inner margin and 5 bipinnate setae in apical position.

P1-P2 with 3-segmented exopods and endopods. P3-P4 with 3-segmented exopods and 2-segmented endopods. P1-P4 praecoxa well developed. P1 (Fig. 15E): praecoxa and coxa with outer spinule row on anterior surface; one posterior row of thin spinules inserted on coxo-basis boundary. Basis with 1 outer spiniform seta and 1 inner spine, with spinule rows along outer margin, between exopod and endopod and at the insertion of inner spine, respectively. Endopod distinctly shorter than exopod, reaching about distal third of exp-2; exp-1 long, about as long as exp-2 and -3 together; exp-1 and -2 with 1 outer spine; exp-3 with 2 outer curved, unipinnate spines, and 1 apical and 1 subapical geniculate setae. Endopod: enp-1 unarmed, about as long as enp-2 and enp-3, wider than enp-2 and enp-3. Enp-2 cylindrical, unarmed. Enp-3 with 1 inner long seta, and 2 apical geniculate setae of different length. Ornamentation as in Fig. 15E.

**Figure 16.**
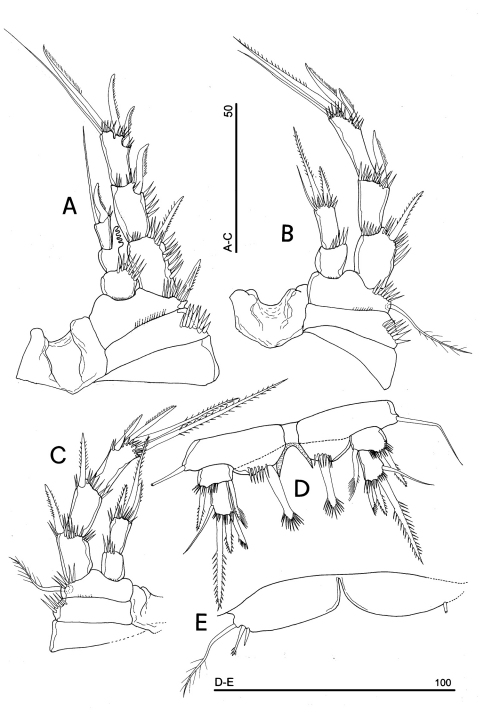
*Parbatocamptus jochenmartensi* Dumont and Maas, 1988 (♂). **A** P2 **B** P3 **C** P4 **D** P5 **E** P6 (scale bars in μm).

P2 (Fig. 16A): praecoxa unornamented, coxa as in P1 and P2. Basis with 1 outer spiniform seta, with spinule rows along outer margin. Exopod distinctly longer than endopod; endopod reaching about the proximal half of exopodal segment 2; exopodal segments of about the same length; exp-1 and -2 with 1 outer spine; exp-3 with 2 outer unipinnate spines, 1 apical fringed seta and 1 subapical long and slender seta. Endopod: enp-1 unarmed; enp 2 transformed, with outer strong comb-like process; enp-3 with 1 transformed spine and 1 long slender and naked seta in apical position. Ornamentation as in Fig. 16A.

P3 (Fig. 16B): praecoxa unornamented; ornamentation of coxa as in P1 and P2. Basis with long outer plumose seta and spinule rows along outer margin and at the insertion of the endopod. Exopod distinctly longer than endopod; endopod reaching about half of exp-2. Exp-1 and -2 with 1 outer spine; exp-3 with 2 outer fringed spines, 1 apical unipinnate seta and 1 subapical long and naked seta. Endopod: enp-1 unarmed; enp-2 with 2 apical spinulose setae and 1 subapical thin and naked seta. Ornamentation as in Fig. 16B.

P4 (Fig. 16C): slightly smaller than the other swimming legs, praecoxa present, unornamented; coxa with outer spinule row, one posterior row of thin spinules inserted on coxo-basis boundary; basis with outer plumose seta, and spinule rows along outer margin and at the insertion of endopod; exopod distinctly longer than endopod. Exp-1 and -2 with 1 outer bipinnate spine; exp-3 with 2 outer unipinnate spines and 2 apical bipinnate setae of different length. Endopod: enp-1 unarmed; enp-2 with 1 spine and 1 seta in apical position, and 1 subapical short unipinnate seta. Ornamentation as in Fig. 16C.

P5 (Fig. 16D): free, with clear articulation to P5-bearing somite; right and left legs distinct, trace of intercoxal sclerite present but hardly observable, with coxo-basis protrusions; exopod discrete, 2-segmented, segment 1 with 1 outer spine and 1 inner seta distally fringed; segment 2 with 4 elements: 1 outer slender and naked seta, 1 apical long spine, 1 medial short spine and 1 short seta distally crested; endopod incorporated to basis forming a baseoendopod, trace of original segmentation still recognizable on posterior surface (Fig. 16D); rudimentary endopod 1-segmented, bearing 1 strong spiniform element, crested on its distal margin. Basipodal outer seta slender and naked.

P6 (Fig. 16E): well developed, symmetrical, right and left legs distinct, deep medial incision marking boundary between legs; armature consisting of 1 outer long, bipinnate seta and two inner short spines, the innermost the shortest.

#### Neophyllognathopus

Galassi & De Laurentiis gen. n.

urn:lsid:zoobank.org:act:D06A6D6C-129B-4142-8C3C-B220A4E7CFAD

http://species-id.net/wiki/Neophyllognathopus

##### Diagnosis.

Phyllognathopodidae. Habitus slightly dorsoventrally flattened with no clear demarcation between prosome and urosome. Integumental dorsal window on cephalosome not confirmed. Integument without surface pits, moderately sclerotized. Cephalosome rounded; rostrum elongate, clearly articulated to cephalosome. Cephalosome and both thoracic and abdominal somites with cuticular ornamentation represented by dorsal sensilla. First pedigerous somite free. Hyaline frills of cephalosome, somites bearing P1-P4 plain both dorsally and ventrally. P5-bearing somite with large paired pores laterodorsally. Sexual dimorphism in antennule, P5, P6, urosomal segmentation and ornamentation, and morphology of anal operculum. Female first and second abdominal somites fused forming the genital double-somite. Female urosomal segments with plain hyaline frills ventrally. Female genital apparatus simplified; copulatory pore located at the end of the proximal third of the genital double-somite. Seminal receptacles laterally located and condensed close to the lamellar sixth legs. Male urosome with different arrangement of hyaline frill ornamentations: urosome consisting of 6 segments, second urosomite with indented hyaline frill, third and fourth urosomites with deep ventral sockets; socket on third urosomite plicate, with smooth free distal margin, and 2 setules laterally inserted close to the socket opening; socket on fourth urosomite with free distal margin ornamented by strong and long spinules, covering the opening; fifth urosomite with indented hyaline frill. Anal somite with paired sensilla on dorsal side. Anal operculum protruding free distal margin of anal somite and extruded in strong spinular processes. Sexual dimorphism in the number of spinular processes of anal operculum (3 in females vs. 4 in males; and, in general, anal operculum in male more armed than in female). Caudal rami sub-quadrate, with incomplete setal pattern (6 setae). Dorsal seta inserted on distal third of caudal ramus. Antennule: 8-segmented in female, basically 9-segmented in male; geniculation between segments 7 and 8; penultimate and last segments, each with suture line marking original segmentation between former segments 8 and 9, and 10 and 11, respectively. Long tube-pores on segments 1 and 2 in both sexes. Antenna: armature of the second endopodal segment as in *Phyllognathopus* and *Parbatocamptus*, consisting of 10 elements. Exopod 1-segmented, with 3 lateral and 2 apical setae. Mandible: mandibular palp biramous, basis with inner spinule row, exopod with 1 apical and 1 inner setae; endopod with 1 inner, 1 subapical and 2 apical setae. Armature of maxillule and maxilla as in *Phyllognathopus*. Maxilliped: phyllopodial, lamelliform, 1-segmented. Clear trace of ancestral 2-segmented condition marked by the presence of outer and inner incisions as in *Parbatocamptus*. Armature consisting of 11 elements: 1 strong spine inserted at inner corner of former segment 1; 4 spines and 1 spiniform short seta inserted along inner margin, 5 bipinnate setae in apical position, armature topology basically referable to that of *Parbatocamptus*.

P1-P3 with 3-segmented exopods and endopods. P4 with 2-segmented exopod and endopod. P1-P3 praecoxa well developed. P1 exopod and endopod of about the same length; P2-P3 endopods shorter than exopods, reaching about tip of exp-2. P4 small - sized, praecoxa missing. Female P5: free, with clear articulation to P5-bearing somite; right and left legs distinct; baseoendopod and exopod coalescent, deep incision marking original segmentation between them; endopodal lobe well developed, elongate, longer than exopodal lobe, rectangular in shape, bearing 1 long pinnate seta, subdistally inserted, close to outer margin and a spinule row apically inserted. Exopodal lobe wide, fully incorporated into baseoendopod; exopodal armature consisting of 4 elements, the outermost bipinnate seta inserted in subdistal position, and three apical elements: 2 spinulose and 1 short setae; basipodal outer seta present. Female P6 rudimentary, each leg defined by a small cuticular lateral plate bearing a short, naked seta with rounded tip. Male P5: free, with clear articulation to P5-bearing somite; right and left legs separate, intercoxal sclerite rudimentary, but still discernible. Basis of each leg expanded, endopod strongly trasformed, consisting of a sclerotized and strong protrusion articulated to basis. Endopodal seta bipinnate, inserted on posterior surface of the endopod, close to its articulation to basis. Exopod distinct, clearly articulated to basis, wide and short, rectangular in shape, representing most part of the free distal margin of each leg; exopodal armature consisting of 6 elements, the innermost spiniform seta curved inward. Male P6: right and left legs distinct but closely adjacent to each other along their medial margin, and symmetrical; each leg consisting of a well developed lamellar plate, with spinule row on the anterior surface; armature consisting of 2 inner spines of different length and 1 outer seta.

##### Type species by monotypy.

*Phyllognathopus bassoti* Rouch, 1972 = *Neophyllognathopus bassoti* (Rouch, 1972), comb. n.

##### Etymology.

The genus name is derived from the type genus *Phyllognathopus* and the Latinised Greek prefix *νέοσ* which means “new”, referring to the new position of *Phyllognathopus bassoti* in the systematics of the family Phyllognathopodidae.

#### Neophyllognathopus bassoti

(Rouch, 1972) comb. n.

http://species-id.net/wiki/Neophyllognathopus_bassoti

Figs 17 [Fig F18] [Fig F19] [Fig F20] [Fig F21] [Fig F22] [Fig F23] [Fig F24] 25

##### Neotype designation.

Female neotype completely dissected and mounted in polyvinyl lactophenol, deposited at the Natural History Museum, London (reg. No. NHM.2008. neotype). Other material: 5 ♀♀ and 3 ♂♂ mounted on slides, 5 ♀♀ and 5 ♂♂ processed for SEM; India, 7 January 1999, Y. Ranga Reddy coll.; 1 ♀, slide code 66/49, 1 ♀, slide code 66/53, 1 ♂, slide code 66/55, Santa Fe, Bantayan island, Pooc, Philippines, V. Cottarelli coll. (see Bruno and Cottarelli 1999, for locality details).

*Neophyllognathopus bassoti* is proposed herein as new combination for *Phyllognathopus bassoti* assigned by Rouch (1972) in the original description to the genus *Phyllognathopus*. According to ICZN (2000), a neotype may be designated when *no name-bearing type specimen (i.e. holotype, lectotype, syntype or prior neotype) is believed to be extant and an author considers that a name-bearing type is necessary to define the nominal taxon objectively* (Article 75.1). Article 75.3 asks also for *qualifying conditions* for the establishment of a neotype; among them: …. *a statement that it is designated with the express purpose of clarifying the taxonomic status or the type locality of a nominal taxon* (Article 75.3.1), and *the author’s reasons for believing the name–bearing type specimen(s) … to be lost or destroyed, and the steps that have been taken to trace it or them* (Article 73.3.4).

The specimens on which Rouch (1972) based the original description of *Phyllognathopus bassoti* no longer exist, and most part of the Rouch’s collection has been lost (Rouch, in litt.). Consequently, the ICZN (2000) recommendation 75A cannot be met, because no extant paratypes or paralectotypes, nor topotypic specimens are available, in order to select among them a neotype. The need to clarify the taxonomic status of this species, which is ranked herein to a new genus, imposed to follow another formal procedure, which, if not completely fulfills the ICZN rules (Article 75.3.6), is accepted by the Code (Article 76.3): *the place of origin of the neotype becomes the type locality of the nominal species-group taxon, despite any previously published statement of the type locality*. Following these arguments, a consistent population from India has been selected to establish the new genus, together with additional material from Indonesia. It is relevant to observe that other researchers have given consensus (Bruno and Cottarelli 1999, Karanovic and Ranga Reddy 2004) on the attribution of both populations to the species *Phyllognathopus bassoti* described by Rouch (1972) from the Lake Wisdom (New Guinea).

##### Neotype locality.

India, Andhra Pradesh, town of Guntur, Brindavan Gardens, domestic water reservoir filled by a freshwater bore well; coordinates: approx. 16°18'N, 80°29'E (see Karanovic and Ranga Reddy 2004, for more details).

##### Description based on the designed neotype.

**Figure 17.**
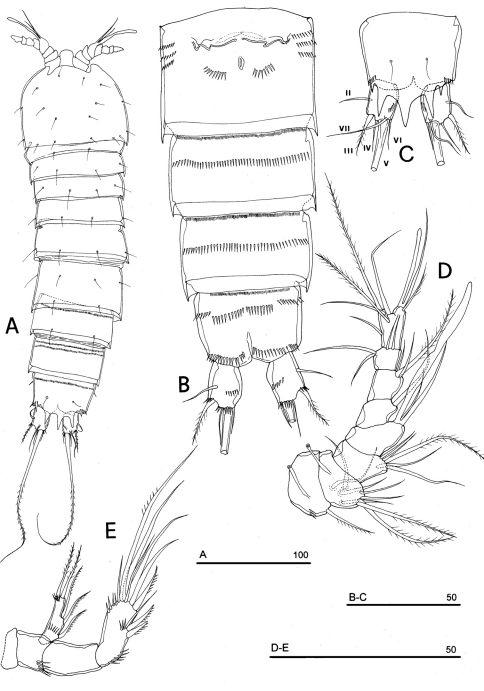
*Neophyllognathopus bassoti* (Rouch, 1972), comb. n. (♀). **A** habitus, dorsal view **B** abdomen, ventral view **C** anal somite and operculum **D** antennule **E** antenna (scale bars in μm).

**Figure 18. F18:**
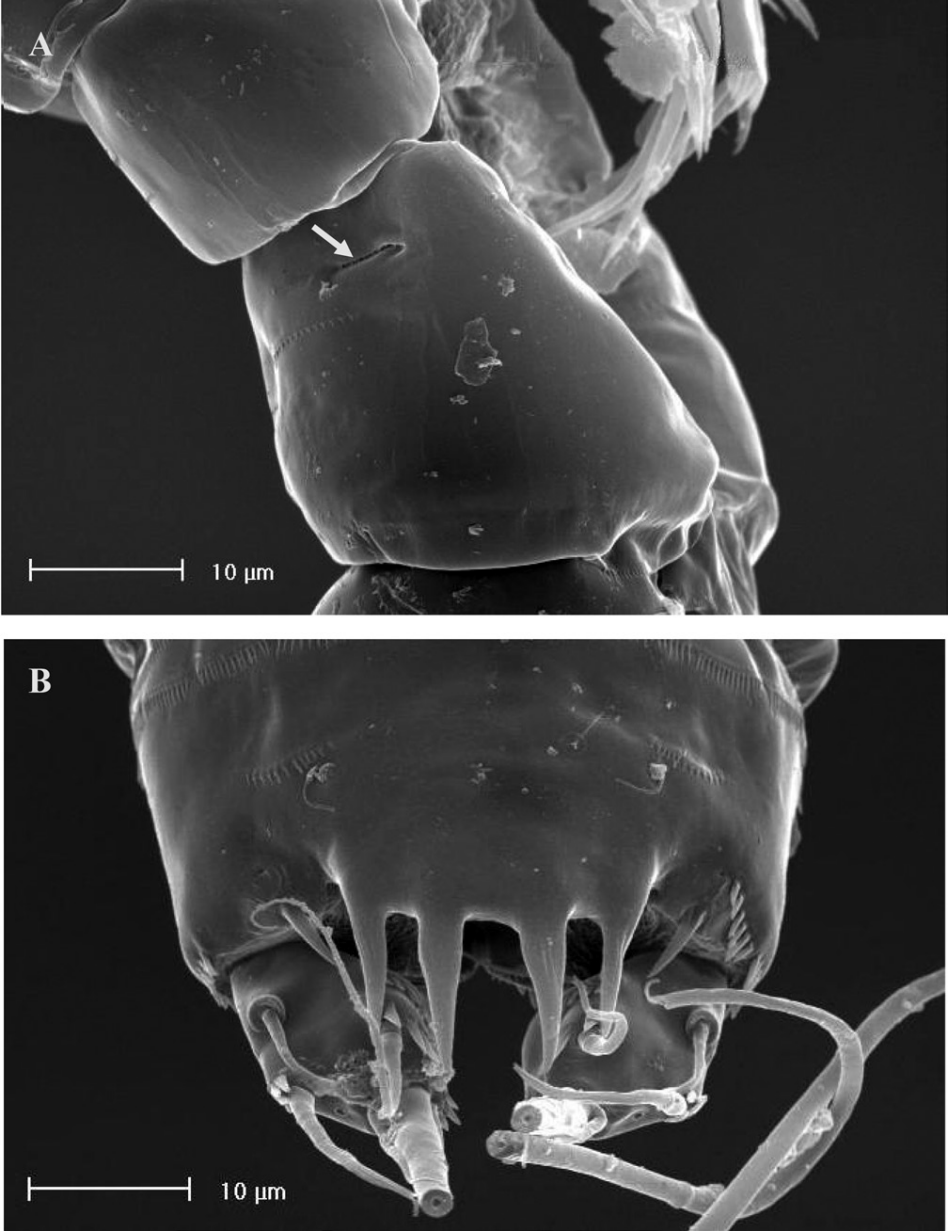
SEM micrographs of *Neophyllognathopus bassoti* (Rouch, 1972), comb. n. (♂). **A** lateral large pore (arrowed) on P5-bearing somite **B** anal somite and operculum.

FEMALE NEOTYPE. Body length, measured from tip of rostrum to posterior margin of caudal rami, 348 µm. Habitus slightly dorsoventrally flattened (Fig. 17A), with no clear demarcation between prosome and urosome. Body depigmented and eyeless. Integumental dorsal window on cephalosome not confirmed. First pedigerous somite free. Integument without surface pits, moderately sclerotized. Cephalosome rounded; rostrum elongate, clearly articulated to cephalosome. Hyaline frills of cephalosome, somites bearing P1-P4 and urosome plain both dorsally and ventrally (Fig. 17 A, B). Cephalosome and both thoracic and abdominal somites (except fourth urosomite) with cuticular ornamentation represented by dorsal sensilla. P5-bearing somite with lateral paired and large pores (Fig. 18A). Female genital field located between first and second third of genital double-somite. Genital apparatus simplified; copulatory pore located at half of genital double-somite. Seminal receptacles laterally located and condensed close to the lamellar sixth legs. Three spinular processes on free distal margin of anal operculum (Figs 17A–C). Caudal rami sub-quadrate, with incomplete setal pattern (6 setae). Dorsal seta inserted close to free distal margin of caudal ramus (Fig. 17C).

Antennule (Fig. 17D): consisting of 8 segments, segments 1 and 2 with long tube-pores. Armature formula: 1-[1], 2-[8], 3-[5], 4-[1 + (1 + ae)], 5-[1], 6-[3], 7-[4], 8-[6 + (1 + ae)]. Aesthetasc on segment 4 very large and long, well overreaching the last antennulary segment.

Antenna (Fig. 17E): exopod and armature of the second endopodal segment as in *Phyllognathopus* and *Parbatocamptus*. Exopod 1-segmented, with 3 lateral and 2 apical setae.

**Figure 19. F19:**
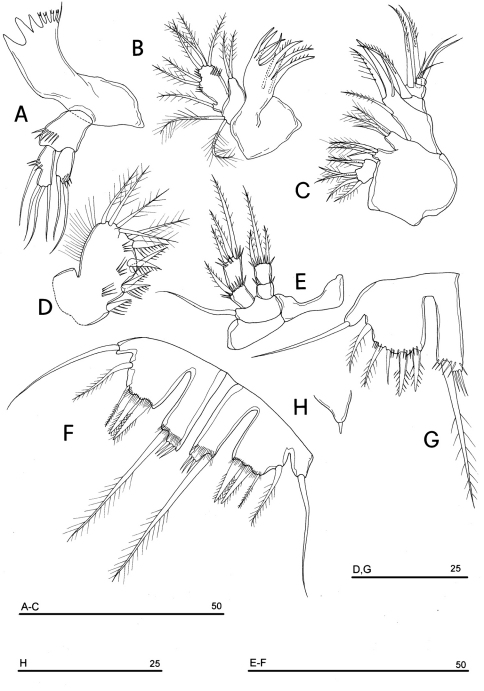
*Neophyllognathopus bassoti* (Rouch, 1972), comb. n. (♀) **A** mandible **B** maxillule **C** maxilla **D** maxilliped **E** P4 **F** P5 **G** P5 (anomaly) **H** P6 (scale bars in μm).

Mandible (Fig. 19A): mandibular palp biramous, basis with inner spinule row, exopod with 1 apical and 1 inner setae; endopod with 1 inner, 1 subapical and 2 apical setae. Armature of maxillule (Fig. 19B) and maxilla (Fig. 19C) as in *Phyllognathopus*.

Maxilliped (Fig. 19D): phyllopodial, lamelliform, 1-segmented. Clear trace of ancestral 2-segmented condition marked by the presence of both outer and inner incisions. Armature consisting of 11 elements: 1 strong spine inserted at inner corner of former segment 1; 4 spines and 1 spiniform short seta inserted along inner margin, 5 bipinnate setae in apical position, armature topology basically referable to that of *Parbatocamptus*.

P1-P3 with 3-segmented exopods and endopods. P4 with 2-segmented exopod and endopod. P1-P3 praecoxa well developed. P4 praecoxa absent (Fig. 19E). P1 exopod of about the same length of endopod. P2-P3 exopods longer than endopods, endopod not overreaching exp-2, fitting the original description (Rouch 1972) and the subsequent ones (Bruno and Cottarelli 1999, Karanovic and Ranga Reddy 2004).

P5 (Fig. 19F, G): free, with clear articulation to P5-bearing somite; right and left legs separate; baseoendopod and exopod coalescent, deep incision marking original segmentation between them; endopodal lobe well developed, elongate, rectangular in shape, longer than exopod, bearing 1 long pinnate seta, subdistally inserted, close to outer margin, and 2 spinule rows, the proximal one composed by tiny elements, the distal one of long spinules; exopodal lobe well discernible, with armature consisting of 4 (rarely 5 elements, observed in only one female) elements, the outermost seta inserted in subdistal outer position, the remaining ones in apical position; the outer apical seta slender and bipinnate, the remaining two spiniform. Basipodal outer seta present.

**Figure 20. F20:**
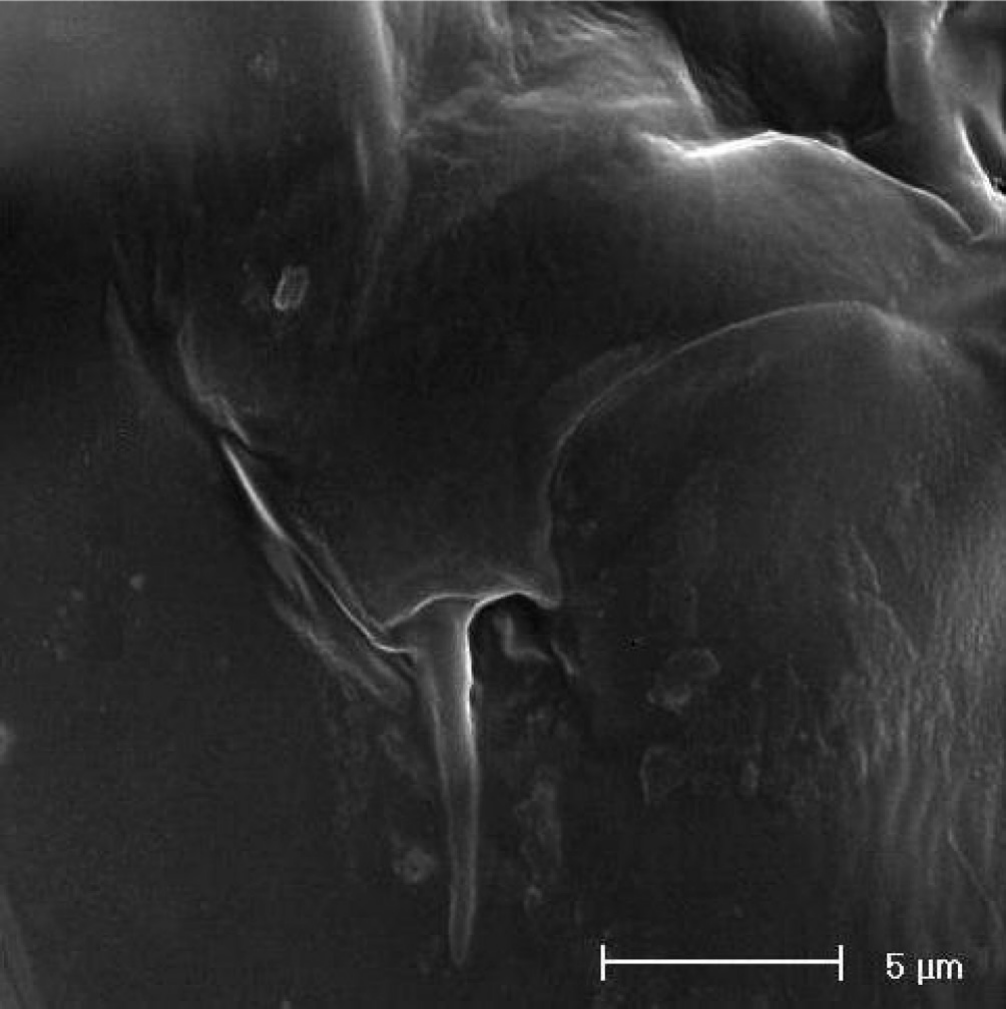
SEM micrograph of *Neophyllognathopus bassoti* (Rouch, 1972), comb. n. (♀): P6.

P6 (Figs 19H, 20): rudimentary, consisting of small paired chitinous lamellar plates not coalescent along medial margin, partially covering seminal receptacles. Armature consisting of 1 short smooth spine with rounded tip on each side.

##### Male.

**Figure 21. F21:**
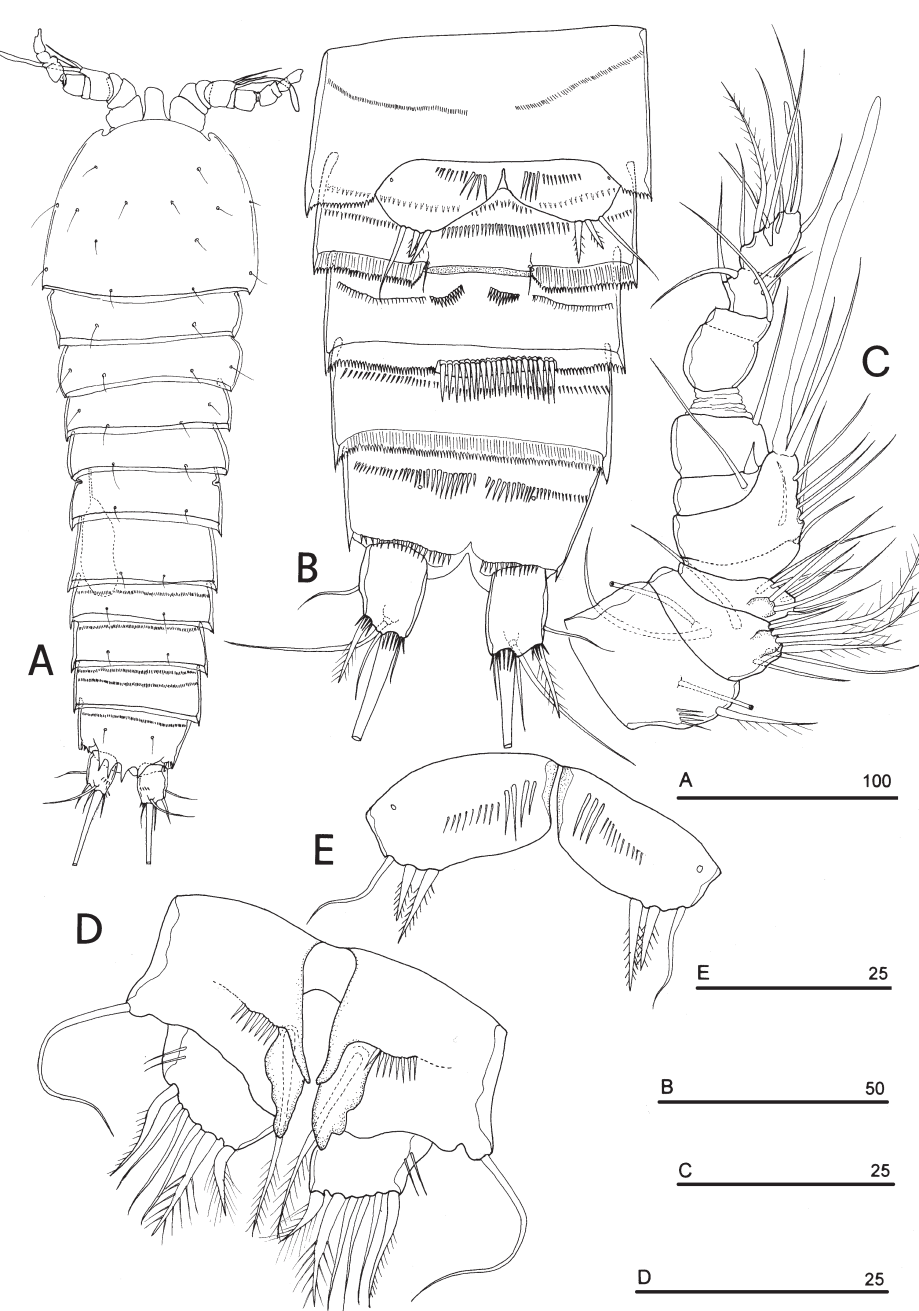
*Neophyllognathopus bassoti* (Rouch, 1972), comb. n. (♂). **A** habitus, dorsal view **B** abdomen, ventral view **C** antennule **D** P5 **E** P6 (scale bars in μm).

**Figure 22. F22:**
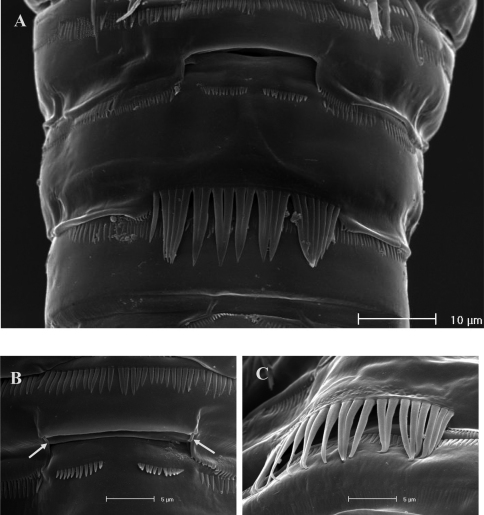
SEM micrographs of *Neophyllognathopus bassoti* (Rouch, 1972), comb. n. (♂). **A** general view of the urosomal sockets **B** plicate socket on third urosomite (paired lateral setules arrowed) **C** socket on fourth urosomite covered by strong spinules.

No marked sexual dimorphism in body size. Body length, measured from tip of rostrum to posterior margin of caudal rami, 335 µm. Rostrum and ornamentation of cephalosome as in female (Fig. 21A). Male urosome consisting of 6 segments (Fig. 21A), third and fourth urosomites with deep ventral sockets (Figs 21B, 22A); socket on third urosomite plicate, with smooth free distal margins, and 2 setules laterally inserted close to the socket opening (Fig. 22B); socket on fourth urosomite with ornamented anterior margin, armed by strong spinules covering the opening (Fig. 22C). Anal somite with paired sensilla on dorsal side. Anal operculum protruding free distal margin of anal somite and extruded in 4 strong spinular processes, rarely 5 (in general anal operculum in males more armed than in females) (Fig. 18B). Antennule (Fig. 21C): basically 9-segmented, geniculation between segments 7 and 8; penultimate and last segments, each with suture line marking original segmentation between former segments 8 and 9, and 10 and 11, respectively. Long tube-pores on segments 1 and 2. Armature formula: 1-[1], 2-[8], 3-[8], 4-[1], 5-[7+(1 + ae)], 6-[2], 7-[1], 8-[1], 9-[10 + (1 + ae)].

**Figure 23. F23:**
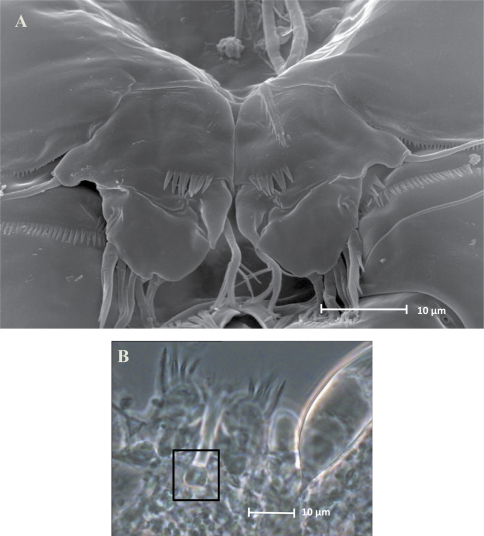
*Neophyllognathopus bassoti* (Rouch, 1972), comb. n. (♂). **A** SEM micrograph of P5 **B** contrast phase micrograph of P5 rudimentary intercoxa (framed).

**Figure 24. F24:**
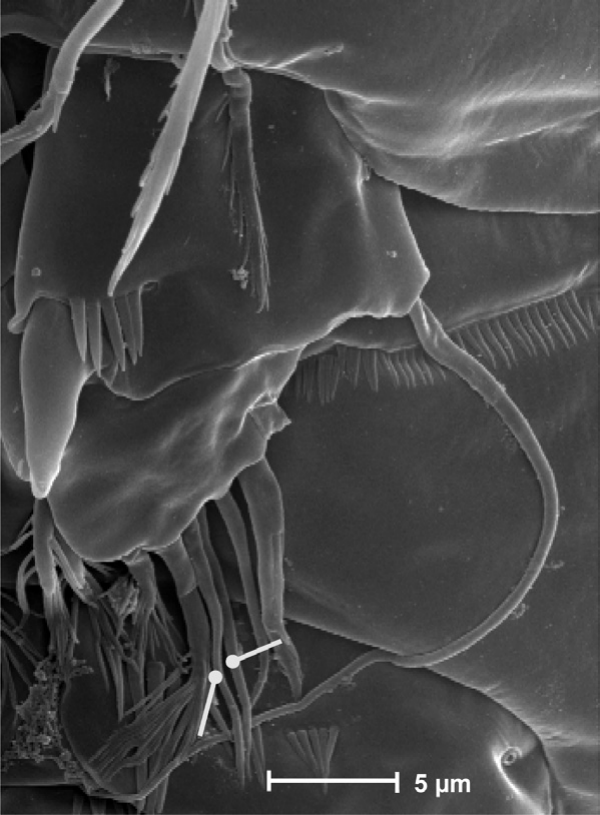
SEM micrograph of *Neophyllognathopus bassoti* (Rouch, 1972), comb. n. (♂) P5, detail (white lines showing setae III and IV, which are closely adherent to each other and hardly discernible as distinct under contrast phase microscope).

**Figure 25.**
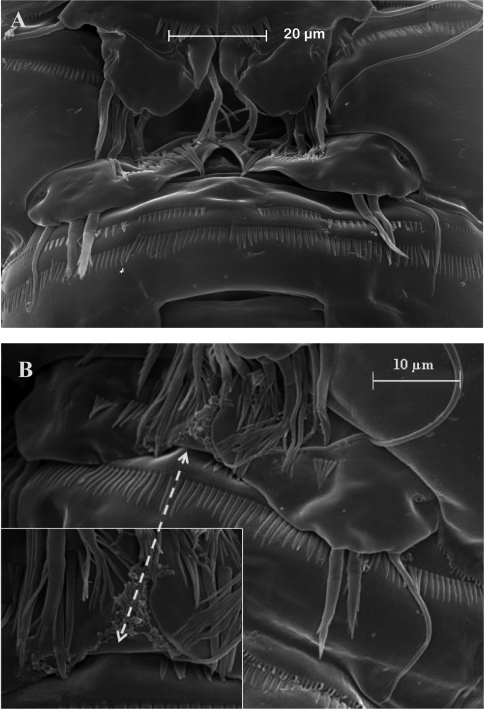
SEM micrographs of *Neophyllognathopus bassoti* (Rouch, 1972), comb. n. (♂). **A** P5 and P6 **B** detail of P6 showing an interconnecting lamella (rudimentary intercoxa?) between right and left legs arrowed.

P5 (Figs 21D, 23A): free, with clear articulation to P5-bearing somite; right and left legs separate, intercoxal sclerite rudimentary but still discernible (Figs 21D, 23B). Basis of each leg well developed, representing most part of each leg; endopod rudimentary, consisting of a sclerotized and strong process articulated to basis. Row of surface spinules inserted near articulation between endopod and basis. Endopodal seta bipinnate, inserted on posterior surface of the endopod, close to its articulation to basis. Exopod distinct, clear articulated to the basis, wide and short, with unusual topology, being placed at the inner free distal margin of basis; exopodal armature consisting of 6 elements, all of which in apical position. Inner spinulose seta short and distinctly curved inward, the remaining setae of about the same length, 2 of which (the second and the fourth, beginning from the inner margin of the exopod) are respectively bipinnate and unipinnate; the remaining 3 smooth and slender, frequently closely adherent to each other and not easily discernible as distinct (Fig. 24). Male P6 (Figs 21E, 25A–B): right and left legs distinct, closely adherent along inner margin, and symmetrical, each leg consisting of a well developed lamellar plate, with some spinule rows on the anterior surface. A membranous lamella is observable between right and left P6 (rudimentary intercoxa?) (Fig. 25B); armature consisting of 2 inner spines of different length and 1 outer naked seta.

## Discussion

According to Dussart and Defaye’s (1990) world catalogue of freshwater harpacticoids, eleven species of *Phyllognathopus* are formally accepted as valid. However, two species, *Phyllognathopus labicauda* Por, 1964 and *Phyllognathopus medius* Por, 1964 must be discounted, since they belong to the genus *Phyllopodopsyllus* T. Scott, 1906 (Tetragonicipitidae), and were erroneously assigned to the genus *Phyllognathopus*. Moreover, *Phyllognathopus coecus* (Maupas, 1892) is still considered a valid species by some authors (Borutzky 1964, Dámian-Georgescu 1970, Dussart and Defaye 1990), whereas it is in reality a junior synonym of *Phyllognathopus viguieri*, as already pointed out by Lang (1948). Dussart and Defaye (1990) also ranked *Phyllognathopus coecus* var. *brevisetosus* (Daday, 1901) and *Phyllognathopus fodinatus* (Ziegelmayer, 1923) as taxa *incertae sedis*, and we have followed their decision since neither Daday (1901) nor Ziegelmayer (1923), strongly criticized by Chappuis (1924), provided the detail required for a correct identity of the taxa. Dussart and Defaye (1990) did not list *Phyllognathopus* sp., reported and partially figured by Dussart (1984) from New Caledonia. According to the author, this species possesses several peculiar morphological characters of high taxonomic significance. Similarly omitted was the species reported from Madras (India) and described by Krishnaswamy (1957) under the name *Phyllognathopus viguieri*, which in our opinion should be transferred to a new species, characterized by the combination of several features, not least the presence of 2-segmented P4 exopods. Unfortunately Krishnaswamy’s type-material no longer exists (Ranga Reddy, pers. comm.), so we admittedly based our conclusions on newly collected material from India (although not from the type-locality). Conflicting opinions on the validity of the individual species of *Phyllognathopus* instigated serious taxonomic confusion. Most workers attempted to solve the problem by synonymising quite orthodoxically the largest number of species and subspecies possible. On the other hand, because of the low standard of published descriptions and the alleged variability observed among (sometimes sympatric) populations, it is not surprising that this taxonomic practice became popular. Most of the supposed morphological variability was found in the morphology and armature of caudal rami and male P5. In different botanical gardens in Great Britain, harbouring different tropical plants, several “morphological types” were observed and discussed by Gurney (1932). In this paper, the author concluded that these forms are to be considered phenotypic variations of the same species *Phyllognathopus viguieri*. Unfortunately, the drawings for each population, although of good quality by contemporary standards, are incomplete for the remaining morphological characters, thus preventing us from attributing a more accurate taxonomic status to the different populations. Moreover, it is not unlikely that they may belong to different species imported from different places together with their host-plants. Chappuis (1940) already reached an analogous conclusion. He observed that the different populations described by Gurney (1932) originated from different botanical gardens in Kew, Oxford and Edinburgh, suggesting that the populations containing males with different P5 baseoendopodal armature and females with different caudal ramus morphology and setation may have been imported together with the tropical plants with which they were associated. For this reason, it is conceivable that they are not native for the country where they were collected. Phytotelmata are the more common habitats for *Phyllognathopus* species. For example, the type-species was originally described from decaying banana trees in Algeria (Maupas 1892). One year later, Mrázek (1893) described *Phyllognathopus paludosus*, but subsequently both Hartwig (1896) and Scourfield (1906) considered it a junior synonym of *Phyllognathopus viguieri*. In particular, Scourfield (1906) argued that Mrázek (1893) based its description of *Phyllognathopus paludosus* on copepodids. Later on, Chappuis (1916) published a comparative table, illustrating major differences between *Phyllognathopus viguieri* (= *Viguierella coeca*) and *Phyllognathopus paludosus*. However, some of the differences listed are doubtful. For instance, in *Phyllognathopus viguieri* the antenna is 4-segmented instead of 3-segmented in *Phyllognathopus paludosus*. In our opinion this difference is based on an observational error, i.e. the failure to recognize the boundary between the small basis and the proximal endopodal segment. This explains why the exopod is either figured (correctly) at the basis-endopod boundary or (erroneously) halfway of the outer margin of what appears to be an allobasis. Other characters, such as the relative length of caudal rami (more than twice longer than wide in *Phyllognathopus paludosus*, vs. 1.5 longer than wide in *Phyllognathopus viguieri*), the shape of the inner terminal caudal seta V (transformed in *Phyllognathopus viguieri*, vs. normal in *Phyllognathopus paludosus*), the anal operculum (smooth in *Phyllognathopus viguieri*, vs. armed with fine spinules in *Phyllognathopus paludosus*), the inner protrusion (= transformed endopod) of male P5 baseoendopod with seta in *Phyllognathopus paludosus*, vs. 1-segmented cylindrical endopod bearing a transformed seta in *Phyllognathopusviguieri*, seem to support the validity of *Phyllognathopus paludosus*, which is considered a valid species also by Borutzky (1964), Damian-Georgescu (1968), and Barclay (1969). More recently, Chang and Yoon (2007) redescribed both *Phyllognathopus viguieri* and *Phyllognathopus paludosus* from South Korea, and the populations they assigned to the above species show minor differences in relation to the available descriptions. In particular, the Korean *Phyllognathopus viguieri* possesses anal operculum with free distal margin smooth or “*with several minute projections*” (Chang and Yoon 2007: 60), whose nature remains doubtful, since this minor ornamentation is not homologous to the spinules of the free distal margin of the anal operculum of several phyllognathopodids; the male P6 is figured and described as a small protrusion, bearing 3 elements, without any mention to the presence/absence of a continuous lamellar plate connecting, or not, right and left P6; again, this condition should require confirmation, because it has never been reported in other descriptions of *Phyllognathopus viguieri*. Moreover, some discrepancies are also observable between drawings and text descriptions. In particular, the maxilliped of *Phyllognathopus viguieri* is figured with 10 elements, vs. described with 9; the male P5 endopod is figured as distinctly 1-segmented, with an additional element at the insertion of the free endopod with basis (Chang and Yoon 2007: figure 2E) but described as *partly fused with exopod with 1 protuberance bearing about 10 spinules or setules around distal margin* (Chang and Yoon 2007: 60). In the same occasion, the authors described and figured *Phyllognathopus paludosus*. The male P5 does not fit previous descriptions in the morphology and construction of the endopod, and the male P6 is partly figured and described as small protrusion. Unfortunately, all the available descriptions of *Phyllognathopus paludosus* are incomplete, preventing any clear statement and critical assessment of the diagnostic features of this species.

Chappuis (1938) assigned one population from the River Ondo, close to Lake Kibuga (Zaire) to *Phyllognathopus viguieri* without describing or figuring any specimens. This species, which had already been recorded from tropical Africa (Lake Tanganyika) (Gurney 1928) was subsequently reported by Chappuis (1956) from a cave in La Réunion. Again, no text description or figures were provided except for the observation that the female caudal rami were at least twice longer than wide and for the remaining characters both sexes in the population fitted the diagnosis of *Phyllognathopus viguieri*. Barclay (1969) described *Phyllognathopus volcanicus* from New Zealand, which resembles *Phyllognathopus paludosus* in most aspects, especially in the morphology and armature of the caudal rami, and differs from the latter only in the relative length of the exopodal setae of the female P5. In *Phyllognathopus paludosus* (as well as in *Phyllognathopus viguieri*) the longest seta is the third, whereas in *Phyllognathopus volcanicus* it is the fourth.

Chappuis (1928) described a new subspecies from bromeliads on the Island Sumatra (Chappuis 1928, 1931), named *Phyllognathopus viguieri menzeli* (Chappuis, 1928) [as *Viguierella coeca menzeli* Chappuis, 1928], on the basis of the armature and morphology of the male P5 baseoendopod, bearing a well developed inner protrusion, representing the former endopod, plus 1 normal pinnate seta, probably corresponding to the *Viguierella* sp. “Salakform” described by Menzel (1926) from Buitenzorg in Java (Indonesia). Subsequently, Chappuis (1931) supplemented the original description with some details on the morphology and armature of the male P2 distal endopodal segment, which bears a long apical transformed spine, giving more robust support for the validity and possibly specific status of this taxon. *Phyllognathopus viguieri menzeli* has also been reported by Watkins and Belk (1975) from phytotelmata in Guam. Their identification was supposedly based on the close resemblance of the male P5 with that originally described by Chappuis (1928) but direct comparison of the respective drawings failed to reveal such close similarity. In the original description the baseoendopodal seta is normally built and setiform, whereas in the material from Guam it is drawn as a large and stout element, not dissimilar in ornamentation from the inner protrusion (transformed endopod), suggesting that they are not homologous. Our examination of the specimens from Guam, on which Watkins and Belk (1975) based their assignment, revealed a quite different situation: 1) male P5 endopod identical to that of *Phyllognathopus viguieri* (1-segmented endopod bearing a large and short leaf-like transformed seta); 2) male P5 exopod wider than in *Phyllognathopus viguieri* but with identical armature; 3) no trace of transformed seta on male P2 enp-3; 4) anal operculum armed with spinules. These observations definitively confirm that the specimens from Guam cannot be assigned to *Phyllognathopus viguieri menzeli*, and they more likely represent a new species, closely related to the nominotypical species *Phyllognathopus viguieri*. Jakubisiak (1929) described *Viguierella coeca parvula* (= *Phyllognathopus viguieri parvulus* (Jakubisiak, 1929)) from mosses in Poznam (Poland). This subspecies differs from the nominotypical species by the smaller body size (288 µm), and the different P5 morphology in both sexes. Unfortunately, this subspecies was insufficiently described and not figured at all in the original description, although some drawings were provided in a subsequent publication (Jakubisiak 1931). In this paper, the author recognized some similarity with the P5 of *Phyllognathopus fodinatus*, but the description and figures of this species by Ziegelmayer (1923) are vague and erroneous in several aspects. For instance, Ziegelmayer’s (1923) fig. 7 represents the male P5 and not the mandible as cited in the legend. This male P5 shows the outer basal seta arising from the exopod, and the baseoendopods being coalescent, displaying no discernible trace of armature, apart from some tiny setules along the free distal margin. Interpreting the swimming leg setation pattern is a most intractable issue because it is impossible to distinguish between ornamentation and armature elements. Lang (1948), being unable to resolve the taxonomic confusion, synonymised several species and subspecies with *Phyllognathopus viguieri*, namely *Phyllognathopus coecus* (Chappuis, 1916), *Phyllognathopus coecus menzeli* (Chappuis, 1928), *Phyllognathopus coecus parvulus* (Jakubisiak, 1929), *Phyllognathopus paludosus*, *Phyllognathopus coecus brevisetosus* (Daday, 1901), *Phyllognathopus fodinatus*, and *Phyllognathopus chappuisi* Delachaux, 1924, claiming the high morphological variability of this species as a reflection of its ecological plasticity.

Chappuis (1940) (not cited by Lang 1948), in his description of *Phyllognathopus insularis* Chappuis, 1940 from mosses collected in the subantarctic Marion Island (Southern Indian Ocean), lent support to the taxonomic validity of *Phyllognathopus chappuisi*, originally described by Delachaux (1924) from a similar habitat in Surinam (South America). Delachaux figured only the antennules, antennae and P5 (all based on a single male provided by Chappuis). Chappuis (1924) himself pointed out the 2-segmented condition of both rami of the P4 and figured this appendage and the P5 in a subsequent paper (Chappuis 1940). With the discovery of this clear-cut character, the systematics of the genus *Phyllognathopus* started to be viewed in a different light. Some characters were considered to have a more robust taxonomic significance than others, but for most morphological features such significance remained obscure. Chappuis (1940) for the first time recognized the importance of P4 exopodal segmentation in assessing the taxonomic position and status of *Phyllognathopus* populations. Both *Phyllognathopus chappuisi* and *Phyllognathopus insularis* share a 2-segmented P4 exopod, together with the widespread 2-segmented endopod, with the distal segment bearing the 3 basic elements. They differ in the arrangement and armature of the male P5 baseoendopod, the inner protrusion (transformed endopod) being accompanied by 1 normal seta in *Phyllognathopus insularis*, but being completely absent in *Phyllognathopus chappuisi*. The male P4 shows an identical setation pattern in both species, differing otherwise only in the relative length of the armature elements, and in the length of the distal exopodal segment (being slightly longer in *Phyllognathopus insularis*). The synonymy proposed by Karanovic and Ranga Reddy (2004) between these species is not strongly supported by evidence and the observed differences were probably underestimated, following Lang (1948)’s practice. (Božic (1965, 1966) started to adopt a biological approach to the taxonomy of *Phyllognathopus* by demonstrating that superficially similar forms did not interbreed and consequently individual species boundaries are much narrower than traditionally believed, necessitating the re-instatement of some taxa previously considered as mere “forms”. He also stressed the necessity to re-validate the taxonomic significance of some morphological characters. On this basis, he described *Phyllognathopus camptoides* Božic, 1965 collected from dead wood on a forest floor near a pond in Gabon, and *Phyllognathopus paracamptoides* Božic, 1968 from mosses in La Réunion (Božic 1968). In regard to *Phyllognathopus camptoides*, Karanovic and Reddy (2004) considered this species junior synonym of *Phyllognathopus chappuisi*, basing their conclusion on the description of *Phyllognathopus cf. camptoides* given by Defaye and Heymer (1996) for a phyllognathopodid population collected fromthe soil cover of a shaded forest in Irangi (Zaire), and arguing that these authors “found enough variability to synonymise these three species” (Karanovic and Ranga Reddy 2004: 131) (i.e. *Phyllognathopus chappuisi*, *Phyllognathopus insularis* and *Phyllognathopus camptoides*). Actually, *Phyllognathopus cf. camptoides* differs from the species described by Božic (1965) by the setation pattern of P5 in both sexes. In the Zaire population the female P5 exopod is armed with 4 setae, vs. 3 in *Phyllognathopus camptoides*; the male P5 exopod bears 5 setae, vs. 6 in *Phyllognathopus camptoides*, and the basis bears the endopodal protrusion accompanied by 1 seta, vs. the same is absent in *Phyllognathopus camptoides*. On the basis of these differences, Defaye and Heymer’s (1996) material could in our opinion be assigned to a different species.

Recently, cross-breeding experiments carried out by Glatzel and Königshoff (2005) strengthened Božic’s results, encouraging a deep re-visitation of the entire putative range of *Phyllognathopus viguieri*, arguing also the potential relevance of differences eventually observed on microcharacters.

A taxonomic dilemma was raised with the description of *Phyllognathopus bassoti*. The most striking morphological features of *Phyllognathopus bassoti*, as reported in the original description given by Rouch (1972), were: 1) the possession of a 2-segmented P4 exopod in both male and female, 2) anal operculum with 3–4 large, long and strong spinules not articulated to free distal margin of anal operculum, and 3) the peculiar morphology and armature of P5 in both sexes. The morphology of P5 is undoubtedly the most distinctive trait of this species, which can easily be distinguished from all other known species of *Phyllognathopus*, *Allophyllognathopus* and *Parbatocamptus*. *Phyllognathopus bassoti* was also recorded from two different wells on Bantayan Island (Philippines) by Bruno and Cottarelli (1999). These populations show some differences with respect to the original description given by Rouch (1972). In particular the female P5 *differs slightly in the shape of the inner lobe of the basipodite, which is shorter with one more seta in our specimens* (Bruno and Cottarelli 1999: 525). Moreover, the male P5 differs in the armature of both exopod and baseoendopod: in the original description from Long Island the exopod bears 5 (as also described and figured by Karanovic and Ranga Reddy 2004 on Indian populations), vs. 6 setae in the Philippine material; the baseoendopod bears a short pinnate seta apparently inserted on the tip of the small endopodal protrusion, incorporated into the baseoendopod in the material from Long Island, vs. in the Philippine specimens it bears two different elements, one long pinnate seta, inserted proximal to the strong inner protrusion. Our re-examination of both Philippine and Indian populations (the latter also by using SEM) revealed a constant exopodal armature consisting of 6 elements, where the innermost spiniform seta is always strong, short and curved inward, and the three outermost setae are naked and slender, two of which closely adherent and superimposed to each other, making difficult their identity (see Fig. 24).

It is therefore not unlikely that differences observed in the relative development and armature of the P5 endopodal lobe are related to different perception of morphological details, because of intrinsic difficulties in observing the mutual position of the inner protrusion (which is a modified endopod) and the relative seta, whose surface insertion is hardly discernible under optical microscopy. Both populations of *Phyllognathopus bassoti* recently described from India (Karanovic and Ranga Reddy 2004) show minor differences in respect to the original description of the species given by Rouch (1972) and more consistent differences in respect to the populations described by Bruno and Cottarelli (1999) from Philippines. We consider the differences observed among populations as reflection of intraspecific variability, and the derived features shared by all the populations strong enough to rank this species to the new genus *Neophyllognathopus*. The other phyllognathopodid genera *Allophyllognathopus* and *Parbatocamptus* are monotypic. *Allophyllognathopus brasiliensis* Kiefer, 1967 is known from the upstream sector (“caatingas”) of the Rio Negro (Brazil) (Kiefer 1967), and *Parbatocamptus jochenmartensi* has been recorded from high-altitude leaf litter in Nepal (Dumont and Maas 1988) (Table 1).

Review of morphological characters in the family Phyllognathopodidae.Comparisons were based almost exclusively on material examined directly and only sporadically on existing descriptions. This course of action was necessary because in most descriptions many morphological details are missing and the drawings are usually so deficient that any comparisons made may be potentially misleading.

**Table 1.** Genera, species and subspecies presently recognized in the family Phyllognathopodidae.

Genus *Phyllognathopus* Mrázek, 1893	
	*Phyllognathopus viguieri* (Maupas, 1892)
	*Phyllognathopus viguieri menzeli* (Chappuis, 1928)
	*Phyllognathopus paludosus* Mrázek. 1893
	*Phyllognathopus insularis* Chappuis, 1940
	*Phyllognathopus chappuisi* Delachaux, 1924
	*Phyllognathopus camptoides* Božic, 1965
	*Phyllognathopus paracamptoides* Božic, 1968
	*Phyllognathopus volcanicus* Barclay, 1969
	*Phyllognathopus inexspectatus* sp. n.
Species and subspecies inquirendae	
	*Phyllognathopus coecus brevisetosus* (Daday, 1901)
	*Phyllognathopus fodinatus* (Ziegelmayer, 1923)
	*Phyllognathopus viguieri parvulus* (Jakubisiak, 1929)
	*Phyllognathopus* sp. (sensu Dussart 1984)
	*Phyllognathopus* sp. (sensu Krishnaswamy 1957)
	*Phyllognathopus cf. camptoides* (sensu Defaye and Heymer 1996)
Genus *Allophyllognathopus* Kiefer, 1967	
	*Allophyllognathopus brasiliensis* Kiefer, 1967 (type-species by monotypy)
Genus *Parbatocamptus* Dumont and Maas, 1988	
	*Parbatocamptus jochenmartensi* Dumont and Maas, 1988 (type-species by monotypy)
*Neophyllognathopus* gen. n.	
	*Neophyllognathopus bassoti* (Rouch, 1972), comb. n. (type-species by monotypy)

Male antennules. Male antennules are primarily 11-segmented, but, at present knowledge, this segmental pattern is not showed by any member of the family, and the derived condition of 10 segments is the most widespread in the family. An 8-segmented male antennule has been reported in *Phyllognathopus viguieri* (cf. Gurney 1932) and *Parbatocamptus jochenmartensi* (cf. Dumont and Maas 1988), and a 9-segmented antennule in *Neophyllognathopus bassoti* comb. n. (see Bruno and Cottarelli 1999, Karanovic and Ranga Reddy 2004). Glatzel and Königshoff (2005) reported a 10-segmented male antennule for *Parbatocamptus jochenmartensi*, probably resulted by counting segments 10 and 11 as distinct; whereas the male antennule of *Allophyllognathopus brasiliensis* is reported by the same authors as 9-segmented, vs. 7-segmented in the original description (Kiefer 1967). These different segmental patterns partly reflect the level of fusion between the penultimate and last segments, which are usually fused together, but at least in some species the boundary between them is still discernible on posterior surface. Our examination of several species and different populations revealed the presence of an additional segment distal to segment 8. In most phyllognathopodids, this short segment is discrete, and was overlooked in past descriptions The setation patterns on individual segments are virtually impossible decipher on the basis of published illustrations due to omission of setation elements. Our comparative analysis suggests that there is a relatively common, conservative setation pattern among populations and species, although the total number of setae counted per each segment may change on the basis of the different fusion patterns observed in different species.

Our re-examination of the segmental patterns of the male antennule revealed that *Phyllognathopus viguieri* possesses a basically 10-segmented antennule, where the penultimate and last segments are still distinct only on posterior surface (giving an incipient 11-segmented antennule) and fused on frontal surface. *Parbatocamptus* *jochenmartensi* shows a 10-segmented antennule, whereas *Neophyllognathopus bassoti* comb. n. displays a 9-segmented antennule, because both segments 8 and 9, and 10 and 11 respectively, are not distinct, probable reflection of heterochrony.

Novel structures were discovered on the antennules in both sexes; in particular, a truncated tubular extension, probably ending in a distal opening, is discernible on the first and second antennulary segments in both males and females. In many harpacticoid families, these segments commonly possess (tube-) pores, as in, for example, the Neobradyidae (Huys 1987), Cylindropsyllidae (Huys and Conroy-Dalton 1993), Leptastacidae (Huys and Todaro 1997) and Ambunguipedidae (Huys 1990), and it is conceivable that the hyaline structures in phyllognathopodids represent tubular extensions of these pores. Similarly, flaccid structures can also be found on other appendages such as the antenna in the genera of the Leptopontiidae where it is expressed as a backwardly directed tubular “seta” on the allobasis (Huys and Conroy-Dalton 1996) and almost certainly represents the external tubular extension of the persisting antennary gland (R. Huys, pers. comm.). An analogous aesthetasc-like structure is found on the maxilla in the Asterocheridae (Siphonostomatoida) where it forms a hyaline extension around the syncoxal exit of the maxillary gland (Huys and Boxshall 1991: Fig. 2.9.21D). Given their transparent nature, it is not surprising that antennulary tube-pores have not been documented before in phyllognathopodid descriptions. Our analysis confirmed their presence in all *Phyllognathopus* species examined, as well as in *Parbatocamptus jochenmartensi* and *Neophyllognathopus bassoti* comb. n., suggesting that this character may well be an autapomorphy for the family. The functional significance of the tubular extensions is as yet unknown but their presence and identical position in adults of both sexes appears to rule out a possible role in mate location or guarding.

Antenna. The antenna of *Phyllognathopus viguieri* has been reported as 4-segmented, vs. 3 segmented in *Phyllognathopus paludosus*, but this different segmentation pattern stems from failure to identify the segment boundary between the basis and the proximal endopod segment, and consequently the correct position of the exopod. In the original description (Mrázek 1893) of *Phyllognathopus paludosus*, the exopod is positioned on what appears to be an allobasis. Chappuis (1916) uncritically accepted this character as a diagnostic difference between *Phyllognathopus viguieri* and *Phyllognathopus paludosus*. The same observational error was made by Dámian-Georgescu (1970) and Borutzky (1964) in their redescriptions of *Phyllognathopus paludosus*. Our re-examination revealed that the antenna is invariably 4-segmented, comprising a coxa, a basis bearing the 1-segmented exopod, and a 2-segmented endopod.

Mandible. The basic structure of the mandible and the setation of the mandibular palp in the type-genus *Phyllognathopus* appear to be identical in all observed populations, the only exception being *Neophyllognathopus bassoti* comb. n., which, according to Rouch (1972), bears one short seta on the basis. In the Philippine and Indian populations of this species, as redescribed and figured by Bruno and Cottarelli (1999) and Karanovic and Ranga Reddy (2004) respectively, as well as in all species of *Phyllognathopus* we have examined, this seta is absent. As a matter of fact, the presence of a true seta is doubtful and requires confirmation as it could have been confused with one of the spinules forming the surface row that is always present in the same position (but it was not figured by Rouch 1972). On this regard, Karanovic and Ranga Reddy (2004) referred to a spinule element in the Indian populations of *Neophyllognathopus bassoti* comb. n.

Re-examination of the male holotype of *Parbatocamptus jochenmartensi* revealed the presence of a well developed pinnate seta on the basis of the mandibular palp (not figured nor described in the original description), accompanied also by the typical spinule row. This observation suggests that the basis is armed in primitive phyllognathopodids. Interestingly, *Parbatocamptus jochenmartensi* also shows two transformed, prehensile setae on the endopod of the mandibular palp, a character already reported by Dumont and Maas (1988), which is a clear autapomorphy of the genus.

Maxillule and maxilla.The structure of the maxilla and maxillule is almost identical in all specimens observed, although comparison of the setation patterns with previously described species is hampered by inconsistencies and deficiencies contained in most descriptions. As regards to the maxilla, number of setae and their topology are identical in all the species examined. In *Parbatocamptus jochenmartensi* the proximal endite is incorporated into the syncoxa and is composed by four lobes vs. the same endite is articulated to the syncoxa and the lobes are only hardly discernible in other members of the family. The maxillule is more conservative in both morphology and setation. The only exception is represented by the maxillule of *Phyllognathopus viguieri*, where the praecoxal arthrite bears 10 elements, whereas in all the other phyllognathopodids it bears 9 elements, being the proximal surface seta (inserted on a small knob) missing.

Maxilliped. The phyllopodial maxilliped is bilobed, with the basal part (syncoxa) fully incorporated in the compound distal part (basis and endopod fused). With regard to armature, a direct analysis of material among all the populations analysed, revealed two different setation patterns, accompanied by a different degree of fusion between the former syncoxa and the baseoendopod. In all the examined populations of *Phyllognathopus* the maxilliped bears 10 elements, and only a rudimentary incision between syncoxa and baseoendopod is still discernible. On the contrary, the primitive distinction between syncoxa and baseoendopod is still retained in *Parbatocamptus jochenmartensi* and *Neophyllognathopus bassoti* comb. n.with both inner and outer incisions marking original segmentation; moreover, an additional element (a robust stout spine) is inserted along inner side of the boundary syncoxa-baseoendopod, suggesting this condition as primitive within the family.

Integumental windows patterns.The dorsal integumental window (“nuchal organ”) on the cephalosome has been reported only for *Neophyllognathopus bassoti* comb. n. by Bruno and Cottarelli (1999). Our SEM observations failed to confirm its presence in *Phyllognathopus viguieri*, as well as in *Neophyllognathopus bassoti* comb. n. SEM analysis revealed only a rounded globose structure in *Phyllognathopus viguieri* with the same topology of the nuchal organ; no solution of continuity of the cuticle is observable in all the specimens analysed.

Swimming legs P1-P4.Female P1-P3 are relatively identical in both morphology and armature in virtually all species and populations of *Phyllognathopus*. The only remarkable difference is observable in members of the family characterized by a 3-segmented P4 exopod. In particular, in *Phyllognathopus viguieri* and related species, the P4 exp-2 lacks the outer spine in all the examined populations, whereas it is present in *Parbatocamptus jochenmartensi* and absent in *Allophyllognathopus brasiliensis*. Another exception refers to a population of *Phyllognathopus viguieri* collected from Lake Léman (Switzerland) by (Dussart (1966, 1967) in which the female P3 exp-3 has been reported with 3 elements only, whereas in all other descriptions it consistently shows 4 elements (2 outer spines and 2 apical setae). This difference was first highlighted by Van de Velde (1974) in her description of *Phyllognathopus viguieri* from Lochristi (Belgium). Contrary to Dussart (1966), our re-examination of material from Lake Léman revealed the presence of 4 elements on the distal segment of P3 exopod.

The major differences between members of the family are found in the segmentation of the P4 exopod which can show three different patterns: 1) 3-segmented; 2) distinctly 2-segmented; or 3) 1-segmented with (*Phyllognathopus paracamptoides*) or without (*Phyllognathopus* sp. *sensu* Dussart 1984) a surface suture marking the original boundary between proximal and distal segments. In *Allophyllognathopus brasiliensis* the swimming leg segmentation pattern is identical to that of *Phyllognathopus viguieri*. According to Dumont and Maas (1988), *Parbatocamptus jochenmartensi* has 3-segmented P1-P4 exopods, 3-segmented P1 endopod, 2-segmented P2-P3 endopods and 3-segmented P4 endopod. Our re-examination of the male holotype revealed that the legs had been mixed up in the original description, as already supposed by Karanovic and Ranga Reddy (2004). In reality, the P2 has a 3-segmented endopod, whereas P3 and P4 display 2-segmented endopods.

Some variation is also expressed in the segmentation of the P4 endopod (in general, it is 2-segmented, but it was described and figured as 1-segmented in *Phyllognathopus* sp. by Dussart (1984).

By analysing different *Phyllognathopus* populations and species, as well as *Neophyllognathopus bassoti* comb. n., the P4 praecoxa is always absent in both males and females, a character never described or reported for the family, whereas all the remaining swimming legs possess it. The absence of praecoxa is always accompanied by a noticeable reduction in the size of P4, irrespective of the segmentation pattern of this leg. Although the absence of the P4 praecoxa may be a potential synapomorphy of the *Phyllognathopus*-*Neophyllognathopus* lineage, its presence/absence in *Allophyllognathopus* requires confirmation before any phylogenetic inference can be drawn. *Parbatocamptus* still possesses a P4 praecoxa. Interestingly the P4 is large, and the exp-2 still bears 1 outer spine, suggesting that *Parbatocamptus jochenmartensi* possesses the most primitive P4 in the family. Members of the genus *Phyllognathopus* do not display sexual dimorphism in the segmentation of swimming legs. However, some authors have documented transformations of particular setae, especially on the male P2 endopod such as in *Phyllognathopus viguieri* by Gurney (1932: fig. 362) and in *Phyllognathopus viguieri menzeli* by Chappuis (1931: Figs 151–152). Unfortunately, we failed to trace the material on which the above authors based their descriptions, but the direct analysis of the specimens from Guam assigned to *Phyllognathopus viguieri menzeli* by Watkins and Belt (1975) revealed the absence of any kind of transformation of setae in P2 enp-3, as well as in any other of the remaining legs. The potential presence of sexual dimorphism in morphology and/or armature of P3 in *Allophyllognathopus brasiliensis* and of P2 in *Parbatocamptus jochenmartensi* remains a hypothesis, since the female is unknown for both genera.

Fifth legs. The fifth pair of legs is sexually dimorphic in both structure and armature, and sometimes differs among species in the morphology of the exopod, in the degree of fusion between exopod and baseoendopod, in the number of exopodal and baseoendopodal setae, or alternatively, in their relative length. The female of *Phyllognathopus viguieri* shows an exopodal lobe with 4 apical elements, and a baseoendopod with 2 pinnate setae. The exopod is incorporated into the baseoendopod but the right and left legs are distinctly separate. *Phyllognathopus inexspectatus* sp. n. possesses a female P5 quite similar to that of *Phyllognathopus viguieri*, differing in the length of the exopodal setae, all shorter than in *Phyllognathopus viguieri* and, more importantly, in the topology of the outermost exopodal seta which is inserted in a clear outer subapical position. Only a few *Phyllognathopus* species show differences in the number of armature elements (e.g. *Phyllognathopus camptoides*). *Neophyllognathopus bassoti* comb. n.differs from any phyllognathopodid species in the unique morphology of female P5. The female P5 baseoendopod and exopod are coalescent, deep incision marking original segmentation between them; the endopodal lobe is well developed, elongate, rectangular in shape, longer than exopod, bearing 1 long pinnate seta, subdistally inserted, close to outer margin; exopodal lobe well discernible; exopodal armature consisting of 4 elements, the outermost seta inserted in subdistal outer position, the remaining ones in apical position.

The male P5 exhibits much more variation within the family, especially in the structure and armature of the baseoendopod. The most primitive condition is observed in *Phyllognathopus viguieri*, which exhibits a discrete 1-segmented endopod, on which the endopodal seta is terminally inserted. In some *Phyllognathopus* species, as well as in *Allophyllognathopus brasiliensis* and *Neophyllognathopus bassoti* comb. n., the endopod is secondarily transformed in a strong spinular process, sometimes described as incorporated into the basis, but more frequently articulating with it. In *Parbatocamptus jochenmartensi* the endopod is partially fused to basis forming a baseoendopod, although the suture line marking original segmentation is still discernible. In most phyllognathopodid species the endopod bears 1 element, but it was also reported without ornamentation. Despite its variation, no significance has been attributed to this character, in so far that populations displaying different setation patterns have been assigned to the same species.

The male P5 exopod shows different construction and armature among members of the family. The most primitive condition is showed by *Parbatocamptus jochenmartensi*, which retains a 2-segmented exopod; the exopod is distinctly 1-segmented in *Neophyllognathopus bassoti* comb. n., and in *Allophyllognathopus brasiliensis*, and appears as incorporated to the basis in *Phyllognathopus* (see also Glatzel and Königshoff 2005: 145).

Direct examination of the Philippine material assigned to *Phyllognathopus bassoti* by Bruno and Cottarelli (1999) (now *Neophyllognathopus bassoti* comb. n.) and of another morphologically close population of this species from India (Karanovic and Ranga Reddy 2004) revealed new informative characters associated with the male P5. Males of both populations, and presumably also the type-series of *Phyllognathopus bassoti*, share the presence of an intercoxal sclerite joining the fifth legs which is completely absent in the female P5. Following this discovery, several populations assigned to *Phyllognathopus viguieri* were re-examined, as well as all available species with 2-segmented P4 exopods. Unfortunately the type-material of *Phyllognathopus camptoides* and *Phyllognathopus paracamptoides* no longer exists and the slide material of *Phyllognathopus cf. paracamptoides* deposited in the NMHN (Paris) is in a bad condition. evertheless, we had the opportunity to examine two new species with 2-segmented P4 exopods, *Phyllognathopus inexspectatus* sp. n. from Italian ground water and another population from India, which is currently being analysed. By comparing several populations and species, we observed that whereas most species of *Phyllognathopus* show a weakly defined, medial sclerotisation, fully incorporated into the baseoendopod, only the three known populations of *Neophyllognathopus bassoti* comb. n.show a well defined intercoxal sclerite (also figured but not described by Bruno and Cottarelli 1999). *Parbatocamptus jochenmartensi* also seems to possess a P5 intercoxal sclerite, which is less easy to discern.

Sixth legs. The sixth pair of legs has only sporadically been described and solely in males. It is bilaterally symmetrical and bears 3 elements on either side: a long outer seta, presumably representing the original outer basal seta, and 2 spinulose inner setae. The male sixth pair of legs shows three different morphologies: 1) in most species it appears as a hyaline linear and continuous lamella, lacking any trace of the primitive paired state showing distinct right and left legs (as in *Phyllognathopus viguieri*); 2) in a second species-group, the lamella appears medially incised as in some taxa currently under study, and in *Parbatocamptus jochenmartensi*, and 3) in *Neophyllognathopus bassoti* comb. n. the sixth legs are deeply incised forming a more complex structure, where also an interconnecting lamella has clearly been observed (rudimentary intercoxa?).

The P6 has never been described nor observed in females, frequently reported as absent (cf. Karanovic and Ranga Reddy 2004) and it was probably assumed that it was absent in phyllognathopodids. Our comparative study revealed the presence of the P6 in the female, bearing one seta only, which is usually short, robust and naked, and, less frequently, represented by a slender pinnate seta, being either very long or short. We conclude that the P6 is sexually dimorphic, being differently constructed in different species, or species-groups.

Ornamentation of urosome.The ornamentation of the urosome deserves special attention. By comparing several populations of *Phyllognathopus viguieri* *sensu lato*, several other *Phyllognathopus* species, *Neophyllognathopus bassoti* comb. n., and *Parbatocamptus jochenmartensi*, clearly distinct ornamentation patterns could be distinguished, as well as previously unnoticed enigmatic structures located on both dorsal and ventral sides of the male urosome. The more complex structures were observed in *Neophyllognathopus bassoti* using SEM (Fig. 22A–C) A similar ornamentation is presumably present in *Phyllognathopus camptoides*, as figured by Božic (1966: 37, and Fig. 2).

Both male and female urosome show large dorso-lateral pores located on the P5-bearing somite. They are referable to the pores observed in several Ameiridae (Galassi et al. 1999) and Canthocamptidae (Galassi, *pers. obs.*).

Caudal rami.Caudal rami are frequently sexually dimorphic and generally considered polymorphic in the females of *Phyllognathopus viguieri*. Variation in caudal rami morphology and setation pattern has been reported for several harpacticoids (Schminke 1991). Since different morphs of *Phyllognathopus* not infrequently co-occur in the same geographical area, some authors (e.g. Gurney 1932, Lang 1948) considered them as ecophenotypes of the widespread *Phyllognathopus viguieri*. Since Gurney’s (1932) material came from different sites and, more importantly, different botanical gardens harbouring different imported tropical plants, it is highly conceivable that these different populations belong to different species and that caudal rami polymorphism is much more limited than previously assumed, as also observed by Königshoff and Glatzel (2008) in reared populations of *Phyllognathopus viguieri* *sensu lato*. On the other hand, the same authors stressed that the morphology of the posterolateral and inner terminal setae of female caudal rami is *per se* a weak diagnostic character, since species with the same setal morphology do not interbreed.

## Conclusion

The re-examination of type-material and/or topotypes of different phyllognathopodid species revealed a systematic scenario more complicate than supposed. The type species of the genus *Phyllognathopus*, *Phyllognathopus viguieri*, was redescribed in detail, analysing several populations coming from different localities world-wide. Some morphological characters within the genus *Phyllognathopus* revealed taxonomic significance, giving ground for the description of *Phyllognathopus inexspectatus* sp. n. from ground water in Italy. The discovery of new informative phylogenetic characters led also to the proposal of a new genus for *Phyllognathopus bassoti*, namely *Neophyllognathopus bassoti* comb. n.

Re-examination of different populations and species of the genus *Phyllognathopus* led to the conclusion that the mouthparts show virtually no variation in structure and setation, and the few differences observed are usually autapomorphies of individual species (with the exception of the structure and setation of the maxilliped and the absence of a seta on mandibular basis which is a synapomorphy of a wider group of species/genera). The most robust discriminating features between species-groups are the segmentation of the P4 exopod, the general morphology of legs 5 and 6 in both sexes, and the morphology and ornamentation of male urosome. The different segmental patterns of P4 exopods observed among species of *Phyllognathopus* allow the identification of three morphological groups: 1) species with 3-segmented exopod (here defined *Phyllognathopus viguieri*-group, including *Phyllognathopus viguieri*, *Phyllognathopus viguieri menzeli*, *Phyllognathopus viguieri parvulus*, *Phyllognathopus viguieri brevisetosus*, *Phyllognathopus paludosus*, *Phyllognathopus volcanicus*); 2) species with 2-segmented exopod (here defined *Phyllognathopus chappuisi*-group, including *Phyllognathopus chappuisi*, *Phyllognathopus insularis*, *Phyllognathopus camptoides*, *Phyllognathopus cf. camptoides* *sensu* Defaye and Heymer (1996)*, P. viguieri* *sensu* Krishnaswamy (1957), *Phyllognathopus* sp.(Galassi and Fiasca, under study), and *Phyllognathopus inexspectatus* sp. n.; 3) species with 1-segmented exopod (here defined *Phyllognathopus paracamptoides*-group, including *Phyllognathopus paracamptoides* and *Phyllognathopus* sp. *sensu* Dussart (1984)). After an in-depth re-examination of different species and populations we refrain from attributing any phylogenetic validity to these groups. The reason for this decision is twofold: 1) the more derived groups (*chappuisi*- and *paracamptoides*- groups) show only one derived character state in comparison to the other ones; 2) this difference exclusively pertains to the reduction in the number of exopodal segments of P4, probably resulting from heterochrony, such as post-displacement. Moreover, this character appears to be evolutionary labile since, for instance, in the *paracamptoides*-group, the boundary-line between the first and the second segment is still partly expressed, reinforcing the hypothesis that the development of P4 is post-displaced relative to that of the other legs (P5 excluded, development of which appears to be decoupled from that of the swimming legs, as also noticed in the Parastenocarididae by Galassi and De Laurentiis 2004). The paedomorphic origin of the fourth leg is reflected in its small size (compared to the dimension of other swimming legs), the absence of the outer spine on the exp-2 in all members of the family showing a 3-segmented P4 exopod, the absence of the praecoxa (vs. present in P1-P3), and the strong tendency towards a reduction of the segmental pattern in both exopod and endopod. Only *Parbatocamptus* retains a relatively large P4, a well developed P4 praecoxa, and the outer spine on P4 exp-2. Despite its unstable ontogeny, being the only variable appendage within the genus *Phyllognathopus*, specimens with 3-segmented exopods have never been found to co-occur in the same “population” with specimens displaying 2-segmented exopods, suggesting that this character may be a useful discriminant at least at species level. Reductions in swimming leg segmentation should be employed with caution when inferring phylogenetic relationships between species or species-groups, particularly when no other evolutionary novelties accompany such derived character states. Evolution in copepods frequently entails character losses or fusions between segments. Nevertheless, it is not unlikely that such reductions may have occurred independently more than once in the evolutionary history of a family or genus. For example, endopodal segmentation can be highly variable in certain harpacticoid lineages, and it would be unwise to automatically attribute excessive phylogenetic significance to groups of species sharing a derived segmentation pattern (e.g. endopod 2-segmented, 1-segmented or absent at all, vs. the 3-segmented primitive condition) without having other synapomorphies in common. Identical endopodal segmentation patterns could potentially be homoplastic, either as the result of convergence by habitat selection, or of parallelism, due to the fact that the morphological character states in question share a common ontogenetic basis. Consequently, the evolutionary instability of the endopodal segmentation should be considered with great caution in assessing the common ancestry of derived taxa within a given lineage. This situation has already been observed in some harpacticoid families, such as the Ameiridae (Galassi et al. 1999, Galassi 2001, Galassi et al. 2009), where differences in endopodal segmentation originated as result of intrageneric evolution (Lee and Huys 2002).

Exopodal segmentation patterns of swimming legs are generally more conservative than those of endopods, and the explanatory power of derived states may be potentially higher in resolving phylogenetic issues. Within the Harpacticoida, members of the same genus very rarely exhibit different segmental patterns in the exopods of P2-P4. For example, in the canthocamptid genus *Hypocamptus* Chappuis, 1929, *Hypocamptus brehmi* (Van Douwe, 1922) shows P3 and P4 with 3-segmented exopods, whereas *Hypocamptus paradoxus* (Kreis, 1926) has 2-segmented P3 and P4. Similarly, the laophontid genus *Laophontina* Norman & T. Scott, 1905 contains species with 2-segmented (*Laophontina acantha* Noodt, 1955; *Laophontina noodti* Kunz, 1983) and 3-segmented (*Laophontina dubia* Norman & T. Scott, 1905; *Laophontina posidoniae* Fiers, 1986) P4 exopods. In another laophontid genus, *Robustunguis* Fiers, 1992, the type species *Robustunguis ungulatus* Fiers, 1992 possesses 3-segmented P2-P4 exopods but in its congener *Robustunguis minor* Fiers, 1992 these rami are only 2-segmented. Unfortunately, our analysis failed to reveal any congruence between exopodal segmentation patterns and other derived character states of phylogenetic significance, and consequently we were unable to delimit any “natural groups” within the genus *Phyllognathopus*. The *Phyllognathopus chappuisi*-“lineage” almost certainly evolved within the *Phyllognathopus viguieri*-group, although its phylogenetic position remains unresolved. Species belonging to this “lineage” all have the 2-segmented P4 exopod, which may have evolved independently and several times in the evolutionary history of the genus *Phyllognathopus*. However, since these species do not share any other derived character states, the monophyly of the *chappuisi*-“lineage” remains questionable as it may include the species of the *paracamptoides*-group and therefore be paraphyletic. On the other hand, if *Neophyllognathopus bassoti* comb. n. may theoretically enter into the “*chappuisi*-lineage” for the possession of a 2-segmented P4 exopod, this species is defined by a combination of unique apomorphies, most of which discovered in the present study: 1) P4 with 2-segmented exopods; 2) unique morphology of P5 in both sexes; 3) peculiar arrangement of the ornamentation of the hyaline frills on the male urosome ventrally; 6) anal operculum bearing 3–4 long and stout spinules, not articulated to the operculum. It also shows several plesiomorphic character states, such as the presence of an intercoxal sclerite between male fifth legs, the bilobed, separate male sixth legs (with rudimentaruy intercoxa?), the phyllopodial maxilliped with clear trace of articulation between syncoxa and basis-endopod, and an additional spiniform seta on the same maxilliped. Within the Phyllognathopodidae, the new genus *Neophyllognathopus* shows feeble relationships with the genus *Parbatocamptus*, which plausibly is the most primitive genus within the family, showing the most plesiomorphic state of male P5, with 2-segmented exopod, trace of endopod together with the presence of a rudimentary intercoxal sclerite; a deeply incised and well sclerotized male P6, the basis of the mandibular palp bearing one long bipinnate seta, the phyllopodial maxilliped with 11 elements and clear trace of the primitive articulation between syncoxa and basis-endopod, and the presence of P4 praecoxa and the outer spine on P4 exp-2, always absent in all members of the family showing a 3-segmented P4 exopod. *Neophyllognathopus* gen. n. shares with *Parbatocamptus* the identical construction and armature of the maxilliped, and the presence of a rudimentary intercoxa in male P5. Such similarities are however symplesiomorphic and more detailed information about *Parbatocamptus* is required (for instance, its female is unknown) before such a relationship can be corroborated. Pending the arrival of new data (e.g. molecular analysis, Glatzel, *in litt.*), it seems justifiable to maintain the *Phyllognathopus chappuisi*-group and the *Phyllognathopus paracamptoides*-group in the genus *Phyllognathopus*, considering the differences in P4 exopodal segmentation as intrageneric variation, and to assign generic rank to *Phyllognathopus bassoti* by creating the new genus *Neophyllognathopus*.

Among members of the *chappuisi*-group, all defined by 2-segmented exopods and endopods, *Phyllognathopus inexspectatus* sp. n. is easily distinguishable by a P4 enp-2 with only 2 apical elements (vs. 3 in all members of this group), a spinulose free distal margin of anal operculum (vs. ciliate in *Phyllognathopus insularis* and armed with strong spinular processes in *Phyllognathopus camptoides*), caudal rami with posterolateral seta III transformed and subapical (vs. apical and not transformed in *Phyllognathopus insularis*). *Phyllognathopus camptoides*, as originally described by Božic (1965) shows only 3 elements on the exopodal lobe of female P5 vs. the widespread condition of 4 elements in all the remaining members of the *chappuisi*-group. The urosome of *Phyllognathopus camptoides* has been figured with spinulose hyaline frills ventrally (somewhat resembling the ornamentation of *Neophyllognathopus* gen. n., and, to lesser extent, *Parbatocamptus*), vs. the same are plain in the new species. The descriptions of *Phyllognathopus insularis* by both Delachaux (1924) and Chappuis (1940) are so generic that any conclusion is inadequate, apart from the armature of the P4 enp-2, described and figured with 3 elements. The *Phyllognathopus viguieri* described and figured by Krishnaswamy (1957), which, as already mentioned, is in need to be transferred to a different species, enters the *chappuisi*-group, being instantly recognizable as different species by the presence of 3 elements on P4 enp-2 and caudal inner terminal seta not transformed. A spinulose anal operculum is shared by this species and *Phyllognathopus inexspectatus* sp. n. The missing male of *Phyllognathopus inexspectatus* prevents us from further considerations on the interspecific relationships with apparently closely related species.

*Phyllognathopus inexspectatus* sp. n. is defined by the combination of the following morphological characters: maxillule with syncoxal proximal surface seta absent (vs. present in *Phyllognathopus viguieri*); P2 enp-2 without inner seta (vs. present in *Phyllognathopus viguieri*); 2-segmented P4 exopod (vs. 3-segmented in *Phyllognathopus viguieri*); 2 apical elements on P4 enp-2 (vs. 3 elements in *Phyllognathopus viguieri*); female P5 exopod with 3 apical and one distinctly subapical outer seta (vs. 4 apical setae in *Phyllognathopus viguieri*); female P6 with long bipinnate and slender seta (vs. short, naked and with rounded tip in *Phyllognathopus viguieri*); anal operculum with spinules (vs. smooth in *Phyllognathopus viguieri*); inner terminal seta normally shaped (vs. short and with proximal part enlarged in *Phyllognathopus viguieri*).

Ecology and biogeography. Phyllognathopodidae occur in both temperate and tropical areas, and at different altitudes, with high preference for phytotelmata, leaf litter, moist soils, pitcher plants, man-made and altered habitats. More rarely they occur in mosses (Reid 2001) and in abandoned coalmines. They invaded also genuine freshwater habitats, as they are frequently found in epibenthic layers of sediments in ponds, streams and lakes, in hyporheic habitats, as well as in phreatic and karstic groundwater systems. Their potential for dispersal seems to be very high, by both active and passive dispersion mechanisms. The most demonstrable example was reported by Rouch (1972) who collected *Neophyllognathopus bassoti* comb. n. from a small sandy island in Wisdom Lake only 20 months after its formation in the lake. The Karaman-Chappuis (Delamare Deboutteville 1954) method used to take this sample prevents us from assessing more accurately the real ecology of the species. An ecological confusion comes also from Karanovic and Ranga Reddy (2004) which recorded this species from India and considered the speciesstygobiont at Kandukur, and stygophyle at Guntur, on the basis of the different habitats from which both populations have been collected.

At present, it is difficult to speculate about the plesiotypic habitat of the family, but it is not unlikely that epigean, semi-terrestrial habitats represent the ancestral and still preferred environment for the family in temperate and, especially, in tropical areas. Circumstantial evidence supporting this hypothesis is provided by the high likelihood of discovering phyllognathopodids in these habitats world-wide, where they also appear to have their highest abundance and species diversity. How they can survive dehydration during low-water periods is unknown. Resting stages have never been found, and dormancy has not been documented until now.

From a biogeographical point of view, the cosmopolitanism of *Phyllognathopus viguieri* has unjustly been overemphasized as discussed also by Glatzel and Königshoff (2005). It is now obvious, however, that under this name several cryptic species are hiding, sometimes only recognizable on the basis of differences in microcharacters (morphology and ornamentation of anal operculum, ornamentation of urosomites, ornamentation and armature of both female and male P5 and P6). More precise information for discriminating different species will undoubtedly become available with the arrival of molecular data analysis. Among true freshwater populations presently attributed to *Phyllognathopus viguieri*, some differences have been observed, the taxonomic significance of which is still debatable. For this reason, it seems more adequate to discuss the distribution of the *Phyllognathopus viguieri*-group, as defined above in its restricted sense: it is cosmopolitan in distribution, and utilizes different habitats, occurring more frequently in phytotelmata. The *chappuisi*-group consists of epigean forms, predominantly distributed in tropical areas of the Southern Hemisphere, the only exceptions being *Phyllognathopus inexspectatus* sp. n., which is the first and only member of this group described from the Holarctic Region as a whole. *Phyllognathopus inexspectatus* may be classified as a stygobiont, as it was collected from a karstic aquifer. The colonization of ground water by the putative ancestor may have occurred before or during the Quaternary glaciation, when most of the epigean elements disappeared, and only some populations survived in refuge habitats, like ground water. The widespread presence of the *Phyllognathopus viguieri*-group in the Northern Hemisphere is probably linked to post-glacial recolonizations, by both active and passive mechanisms.

The new genus *Neophyllognathopus* shows a disjunct geographical distribution in the Oriental and Australasian regions and it is thus far predominantly restricted to ground water *sensu lato* (subsurface freshwater habitats: this is the case of *Neophyllognathopus bassoti* from Long Island (Papua, New Guinea) and from Philippines and India). Some doubts there are also for *Allophyllognathopus brasiliensis*, collected from the “caatingas” located in arid areas in Brazil. These areas are characterized by xeric vegetation and their hydrological regimes are regulated by intermittent rivers, which exist only during the rainy season.

The geographical distribution of the four defined groups is not of great assistance in corroborating or refuting phylogenetic affinity between or within groups. Members of the same group do not show a common track of distribution. The *Phyllognathopus viguieri*-group is cosmopolitan, and, at present, any speculation about the centre of origin of the group is premature. Members of the *chappuisi*-group are distributed in both Northern and Southern Hemispheres, predominantly in tropical areas with the exception of the geographically disjunct location of *Phyllognathopus inexspectatus*, which is recorded from temperate Europe. Two alternative hypotheses may be proposed: 1) the *chappuisi*-group was widespread in the past, and descended directly from a *Phyllognathopus viguieri*-like ancestral stock, but disappeared from the plesiotypic surface habitats in the Northern Hemisphere as a consequence of the drastic climatic changes linked to the Quaternary glaciations, and survived as relict species in refuge environments (e.g. ground water); 2) members of the *chappuisi-*group may have originated independently in different geographical areas from different surface ancestors closely related to the *Phyllognathopus viguieri*-group. In the first scenario, a common origin is hypothesized for the *chappuisi*-group, which could be considered monophyletic within the *Phyllognathopus viguieri*-group; in the second one, the “lineage” should be considered polyphyletic. The new genus *Neophyllognathopus* established for the *Phyllognathopus bassoti*-lineage seems to be the only one for which a monophyletic origin may be reasonably inferred. Its distribution is thus far limited to tropical India, the Philippines and New Guinea, in both the Oriental and Australasian Regions, a very problematic area from a biogeographical point of view (Lomolino et al. 2006). There is some ground to suppose an ancient origin for this group, and a recent colonization from India-Philippines to Long Island by dispersal events. The ecological preferences of the species refer to groundwater habitats *sensu lato*, especially the ecotonal boundary between surface and subsurface environments (e.g. hyporheic and subsurface alluvial habitats). Against this ecological background, it is not surprising that *Neophyllognathopus* shows some relationships with the genus *Parbatocamptus*, collected from leaf-litter in Nepal.

## Keys to genera of Phyllognathopodidae

**Males**

**Table:** 

1	P5 with 1-segmented exopod and endopod, the latter cylindrical or transformed in a spiniform element	3
2	P5 with 2-segmented exopod; endopod incorporated to basis forming a baseoendopod; endopod boundary marked by rudimentary suture; rudimentary intercoxa present; P6 represented by a deeply incised hyaline lamella	*Parbatocamptus*
3	P3 endopod not transformed and with setae and/or spines normally conformed	*Phyllognathopus*
4	P3 endopod transformed, with aesthetasc-like elements on enp-3	*Allophyllognathopus*
5	P5 with intercoxa, exopod discrete, very large and translated at the inner margin of the basis; right and left P6 distinctly separated; third and fourth urosomites with deep ventral sockets	*Neophyllognathopus* gen. n.

Females*

**Table:** 

1	P5 baseoendopod and exopod coalescent; incision marking original articulation between basis and endopod little pronounced; baseoendopod bearing two elements	*Phyllognathopus*
2	P5 baseoendopod and exopod coalescent; deep incision marking original articulation between basis and endopod; endopod bearing one element	*Neophyllognathopus* gen. n.

*female unknown for *Parbatocamptus* and *Allophyllognathopus*.

## Supplementary Material

XML Treatment for Phyllognathopus

XML Treatment for Phyllognathopus viguieri

XML Treatment for Phyllognathopus inexspectatus

XML Treatment for Parbatocamptus jochenmartensi

XML Treatment for Neophyllognathopus

XML Treatment for Neophyllognathopus bassoti
